# Circulating microvesicles and exosomes in small cell lung cancer by quantitative proteomics

**DOI:** 10.1186/s12014-021-09339-5

**Published:** 2022-01-07

**Authors:** Shona Pedersen, Katrine Papendick Jensen, Bent Honoré, Søren Risom Kristensen, Camilla Holm Pedersen, Weronika Maria Szejniuk, Raluca Georgiana Maltesen, Ursula Falkmer

**Affiliations:** 1 Department of Basic Medical Sciences, College of Medicine, QU Health, Qatar University, 2713 Doha, Qatar; 2 Department of Clinical Medicine, Aalborg University, Aalborg, Denmark; 3 Department of Biomedicine, Aarhus University, Aarhus, Denmark; 4 Department of Clinical Biochemistry, Aalborg University Hospital, Aalborg, Denmark; 5 Department of Health Science and Technology, Aalborg University, Aalborg, Denmark; 6 Department of Oncology, Aalborg University Hospital, Aalborg, Denmark; 7 Translational Radiation Biology and Oncology Laboratory, Centre for Cancer Research, Westmead Institute of Medical Research, Westmead, 2145 Australia

**Keywords:** Small cell lung cancer, Proteomics, Tumor-derived exosomes, Tumor-derived microvesicles, Potential diagnostic markers

## Abstract

**Background:**

Early detection of small cell lung cancer (SCLC) crucially demands highly reliable markers. Growing evidence suggests that extracellular vesicles carry tumor cell-specific cargo suitable as protein markers in cancer. Quantitative proteomic profiling of circulating microvesicles and exosomes can be a high-throughput platform for discovery of novel molecular insights and putative markers. Hence, this study aimed to investigate proteome dynamics of plasma-derived microvesicles and exosomes in newly diagnosed SCLC patients to improve early detection.

**Methods:**

Plasma-derived microvesicles and exosomes from 24 healthy controls and 24 SCLC patients were isolated from plasma by either high-speed- or ultracentrifugation. Proteins derived from these extracellular vesicles were quantified using label-free mass spectrometry and statistical analysis was carried out aiming at identifying significantly altered protein expressions between SCLC patients and healthy controls. Furthermore, significantly expressed proteins were subjected to functional enrichment analysis to identify biological pathways implicated in SCLC pathogenesis.

**Results:**

Based on fold change (FC) ≥ 2 or ≤ 0.5 and AUC ≥ 0.70 (*p* < 0.05), we identified 10 common and 16 and 17 unique proteins for microvesicles and exosomes, respectively. Among these proteins, we found dysregulation of coagulation factor XIII A (Log_2_ FC =  − 1.1, *p* = 0.0003, AUC = 0.82, 95% CI: 0.69–0.96) and complement factor H-related protein 4 (Log_2_ FC = 1.2, *p* = 0.0005, AUC = 0.82, 95% CI; 0.67–0.97) in SCLC patients compared to healthy individuals. Our data may indicate a novel tumor-suppressing role of blood coagulation and involvement of complement activation in SCLC pathogenesis.

**Conclusions:**

In comparing SCLC patients and healthy individuals, several differentially expressed proteins were identified. This is the first study showing that circulating extracellular vesicles may encompass specific proteins with potential diagnostic attributes for SCLC, thereby opening new opportunities as novel non-invasive markers.

**Supplementary Information:**

The online version contains supplementary material available at 10.1186/s12014-021-09339-5.

## Background

Lung cancer is the main cause of cancer-related deaths, and the second and third most prevalent cancer in Europe among men and women, respectively [1]. The main histopathological subtypes of lung cancer are small cell lung cancer (SCLC) and non-small cell lung cancer (NSCLC). SCLC is a neuroendocrine carcinoma that accounts for ~15% of lung cancers and is characterized by an aggressive progression to early metastases [2, 3]. Currently, the diagnosis is based on computed tomography (CT) scan and cytology obtained by fine-needle aspiration (FNA) biopsy from the suspected lesion. While CT scans has a high sensitivity and low specificity due to a high false-positive rate [4], FNA is associated with a risk of complications [5]. The poor prognosis of SCLC patients is partially a consequence of late diagnosis, since two-thirds of patients present at advanced tumor stage at the time of diagnosis [3]. Thus, to minimize delays in diagnosis and improve patient safety, better diagnostic procedures are warranted.

Throughout the years, research has been aimed at finding easily accessible, cost-effective and non-invasive biomarkers in lung cancer [6]. Two proteins, NSE and ProGRP, have been documented as suitable for discriminating between NSCLC and SCLC [7] and it has been suggested that a panel including these markers may improve diagnosis [8]. Despite rigorous investigations, the ideal diagnostic biomarker for SCLC has yet not propertied a place in the clinic.

The emerging field of extracellular vesicles (EVs) has unraveled a novel approach for investigating SCLC. They are secreted by virtually all cells, including cancer cells, and are present in several body fluids, making EVs applicable as non-invasive liquid biomarkers [9]. Broadly, EVs are divided into exosomes (small EVs) and microvesicles (MVs or large EVs), which are continuously released under physiological and pathological conditions. The vesicles are loaded with a specific cargo, including lipids, proteins, and genetic material originating from the parent cell. Thus, the content of EVs may to some extent resemble the molecular profiles of the originating cells [10]. Therefore, the use of EVs may provide a revolutionary tool for investigating SCLC in a clinical setting. Proteomic analysis with discovery-based mass spectrometry (MS) is a relatively new approach for discovering novel biomarker candidates in several cancers. Profiling of EV proteomes using this approach has led to identification of novel diagnostic biomarkers in cancers, including ovarian and prostate cancer [11, 12]. Recent studies have identified exosomal biomarkers with diagnostic potential in NSCLC patients using MS [13, 14]. The current study seeks to explore the proteome dynamics of plasma-derived exosomes and MVs from SCLC patients for the identification of significantly expressed proteins that can add new insights into lung cancer biology and early diagnosis. This is the first study inaugurating the potential role of circulating MVs and exosomes in SCLC diagnosis using quantitative proteomics.

## Methods

### Subject characteristics

This observational prospective study included data and blood samples from patients with SCLC, diagnosed and treated with chemotherapy between March 2015 to September 2017 at the Department of Oncology, Aalborg University Hospital, Denmark. Inclusion criteria were: eligibility to receive chemotherapy consisting of platinum and a topoisomerase inhibitor, histopathologically and/or cytologically confirmed SCLC, measurable disease on CT scans, and blood samples eligible for MS analysis. Exclusion criteria were: prior systemic chemotherapy for lung cancer, concomitant anticoagulation treatment (except aspirin or clopidogrel), active or at high risk of overt bleeding of clinical importance, severe coagulopathy such as haemophilia, severe liver dysfunction with impaired coagulation, acute peptic ulcer, intracranial haemorrhage or surgery in the central nervous system within the last 3 months, treatment with any other investigational agent, and participation in other clinical trials. The clinical data, administration of medications, treatment details, and radiological evaluation were collected at time of diagnosis. Staging of SCLC was based on the 7th edition of the tumor, lymph node, metastasis (TNM) classification of lung cancer [15]. The study was approved by the North Denmark Region Committee on Health Research Ethics (N-20140055), reported to the Danish Data Protection Authority (2018-731-5589) and performed in accordance with the Declaration of Helsinki. All included participants provided written informed consent before enrolment in the study. In addition, age-and gender-matched healthy controls (HCs) from the blood bank at Aalborg University Hospital were used for comparison.

### Sample collection and preparation

Blood samples were collected from HCs and from SCLC patients at the time of inclusion (henceforth referred to as SCLC patients) as well as prior to third cycle of chemotherapy (treated SCLC patients). Blood was drawn from the antecubital vein using a vacutainer blood collection device with a 21-gauge needle (Vacuette, Greiner Bio-One, Austria) and collected in 9 mL 0.105 M (3.2%) trisodium citrate tubes (BD Vacutainer^®^, UK). Platelet-poor plasma was prepared by double centrifugation at 2500×*g* for 15 min at room temperature. Plasma collection was stopped 1 cm above the buffy coat and pellet, respectively, after first and second centrifugation. Subsequently, the plasma isolates were snap-frozen in liquid nitrogen and stored at -80 °C until further analysis.

### EV isolation and preparation for MS analysis

EV isolation was performed from 1 mL plasma with one centrifugation at 20,000×*g* for 30 min at 4 °C using an Avanti J-30i centrifuge with a J A-30.50 fixed-angle rotor with a k-factor 280 (Beckman Coulter, Brea, CA, USA). The supernatant from the initial spin of the 20 K pellet was used to prepare the 100 K pellet (100,000×*g* for 1 h at 4 °C). Succeeding the initial centrifugation step for each pellet preparation, the resultant EVs were washed in 1 mL phosphate-buffered saline filtered by a 0.22 µm filter. The final enriched 20 K (microvesicles; large EVs) and 100 K (exosomes; small EVs) samples were resuspended in 20 µL filtered phosphate-buffered saline prior to MS analysis. The samples were lysed and solubilized in 5% sodium dodecyl sulfate containing 50 mM triethylammonium bicarbonate, pH 7.55. Alkylation and tryptic digestion were performed using S-TrapTM Micro Spin Columns (Protifi, NY, USA) essentially as previously described [16]. Proteins were cleaved using PierceTM Trypsin protease, MS Grade (Thermo Fisher Scientific, Waltham, MA, USA) and peptide concentrations were measured by fluorescence using an EnSpire microplate reader (Perkin Elmer, Waltham, MA, USA). Samples were resuspended in 0.1% formic acid and injected with an amount of 1 µg in case of 20 K sample and 0.75 µg in case of 100 K sample.

### Label-free quantitative nano liquid chromatography–tandem mass spectrometry analysis

The peptides from 20 and 100 K preparations were analysed on a nano liquid chromatography-tandem mass spectrometry platform consisting of an Ultimate 3000 and an Orbitrap Fusion Tribrid instrument from (Thermo Scientific Instruments, MA, USA) as previously described [17]. Samples were run in technical duplicates. Due to technical difficulties, two HCs from the 20 K group and two SCLC samples from the 100 K group could not be analysed. All in all 284 raw files were generated, 142 20 K raw files and 142 100 K raw files. The mass spectrometry proteomics data have been deposited to the ProteomeXchange Consortium [18] via the PRIDE [19] partner repository with the dataset identifier PXD028944 for the 20 K data and PXD028885 for the 100 K data.

### Protein identification and quantification

Protein identification and label-free quantification (LFQ) were performed in two different searches, using the EV raw files against the human database from Uniprot (downloaded 09/02/2020 for 20 K and 10/08/2019 for 100 K) and using MaxQuant version 1.6.6.0 (Max Planck Institute of Biochemistry, Martinsried, Germany) for LFQ analysis [20]. The number of entries in the Uniprot Homo sapiens databases were 42,427 (downloaded 10/08/2019) and 48,918 (downloaded 09/02/2020). Carbamidomethyl (C) was used as fixed modification, and the false discovery rate for peptide-spectrum matches, protein, and site were each set at 1%. The maximum number of missed cleavage sites was 2. The mass tolerance for precursor ions was 20 ppm for the first search and 4.5 ppm for the main search. The mass tolerance for fragment ions was 0.5 Da. The minimum ratio count for LFQ was set to 1. Tandem mass spectrometry was required for LFQ comparisons. For quantification of proteins, unique and razor peptides, unmodified and modified with oxidation (M) or acetyl (protein N-terminal) were used. The function match between runs was used, reverse sequences were used for decoy search, and contaminant sequences were included in the search. The analysis in MaxQuant included samples from HCs, SCLC patients, and treated SCLC patients, however, the treated samples are excluded in the statistical analyses.

### Statistical analysis

LFQ values for identified proteins were filtered in Perseus version 1.6.10.50 (Max Planck Institute of Biochemistry, Martinsried, Germany) [20] by the exclusion of potential contaminants, reverse sequences, and proteins only identified by site. A minimum of 2 unique peptides was needed for successful identification. LFQ values were Log_2_ transformed and the mean of technical replicates was used for further analysis. Data distributions were assessed through histograms. Proteins were required to have 70% valid values in at least one group. A Venn diagram (Venny 2.1) [21] was used to investigate proteins common and unique for each group and identified proteins were matched to the top 100 identified proteins from the EV databases Vesiclepedia [22] and ExoCarta [23] (both databases downloaded 03/12/2020).

Data were presented as mean and standard deviations (mean ± SD). Trends in samples were assessed using unsupervised principal component analysis (PCA) on autoscaled data. Differentially expressed proteins were identified between healthy and diseased individuals using a Student’s t-test. Proteins were considered statistically significantly expressed if p < 0.05 and Log_2_ fold change (FC) ≥ 1 or ≤ -1 and were visualized through volcano plots. Comparisons of protein expressions were depicted using raw LFQ values. Significantly expressed proteins presented in Table S4 were subjected to enrichment analysis and annotated with significant gene ontology biological process (GOBP) terms using the functional annotation clustering analysis by The Database for Annotation, Visualization, and Integrated Discovery (DAVID) version 6.8 [24, 25].

**Fig. 1:**
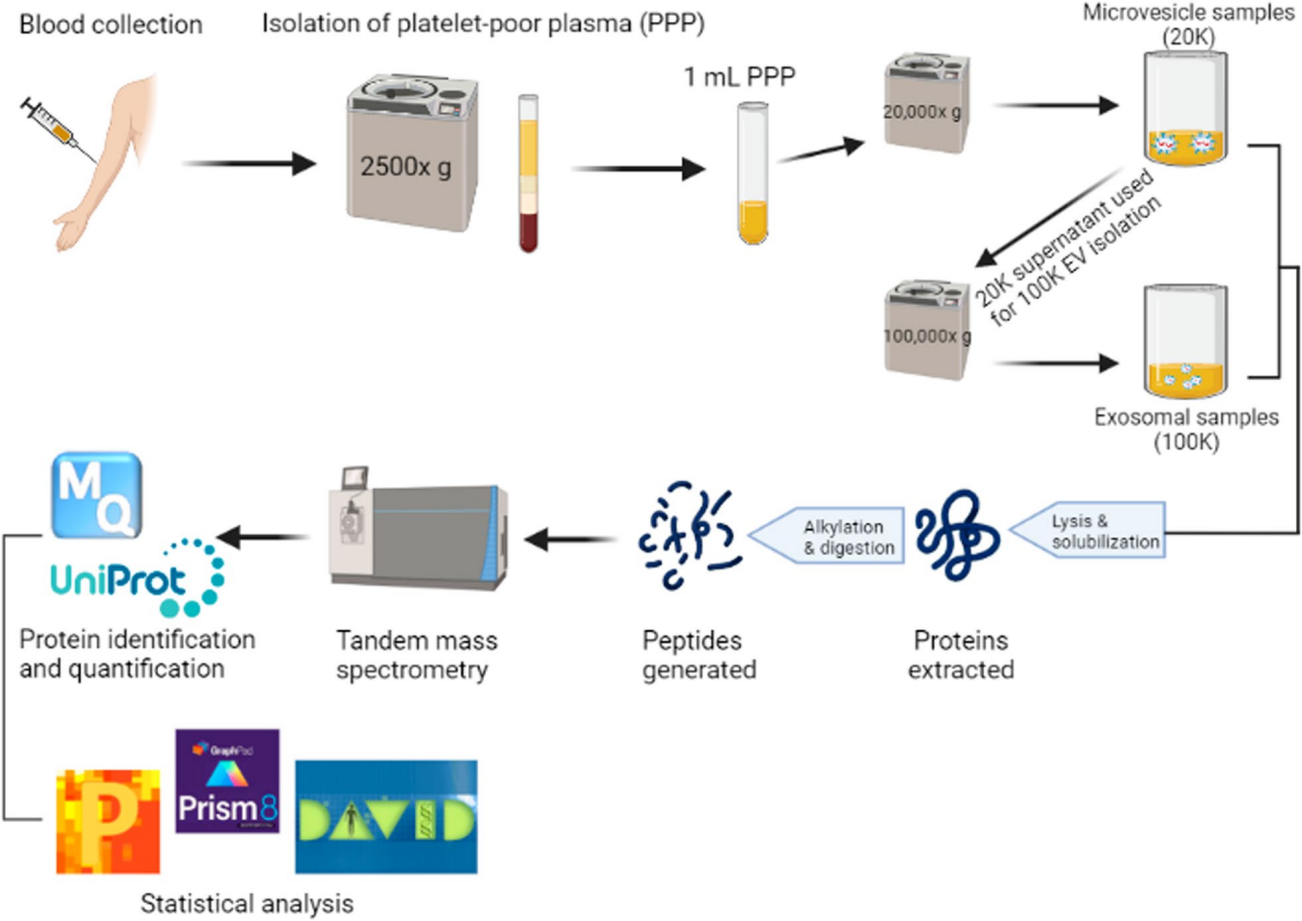
Methodological workflow. The figure was created with BioRender.com

IBM SPSS Statistics 26 (SPSS, Chicago, IL, USA), MATLAB (R2017b, MathWorks, Natick, MA, 24 USA), and GraphPad Prism 8.4.3 (GraphPad Software, La Jolla, CA, USA) were used for statistical analysis. Figure 1 below provides an overview of the sample collection, EV isolation, MS characterization, and statistical analyses of the enriched vesicles.

## Results

### Characteristics of study populations

During the study period, 24 SCLC patients fulfilled the inclusion criteria and were enrolled in the study. A total of 24 matching individuals were enrolled as HCs. Gender and age distributions were balanced among individuals. More than 90% of the patients were diagnosed with advanced stage disease (Table 1).

**Table 1:** Demographics and patient characteristics of the study population

Study characteristics for SCLC patients and healthy controls		
	SCLC patients	Healthy controls
	*N* = 24	*N* = 24
Demographics		
Sex (male/female, *N*)	12/12	12/12
Mean age (± SD)	67 ± 7	63.3 ± 3
Patient characteristics		
TNM stage, N (%)		
IIB	1 (4)	
IIIA	6 (25)	
IIIB	3 (13)	
IV	14 (58)	

*SCLC* small cell lung cancer, *N* number of patients, *SD* standard deviations

### Proteomic analysis of circulating microvesicles and exosomes

**Fig. 2:**
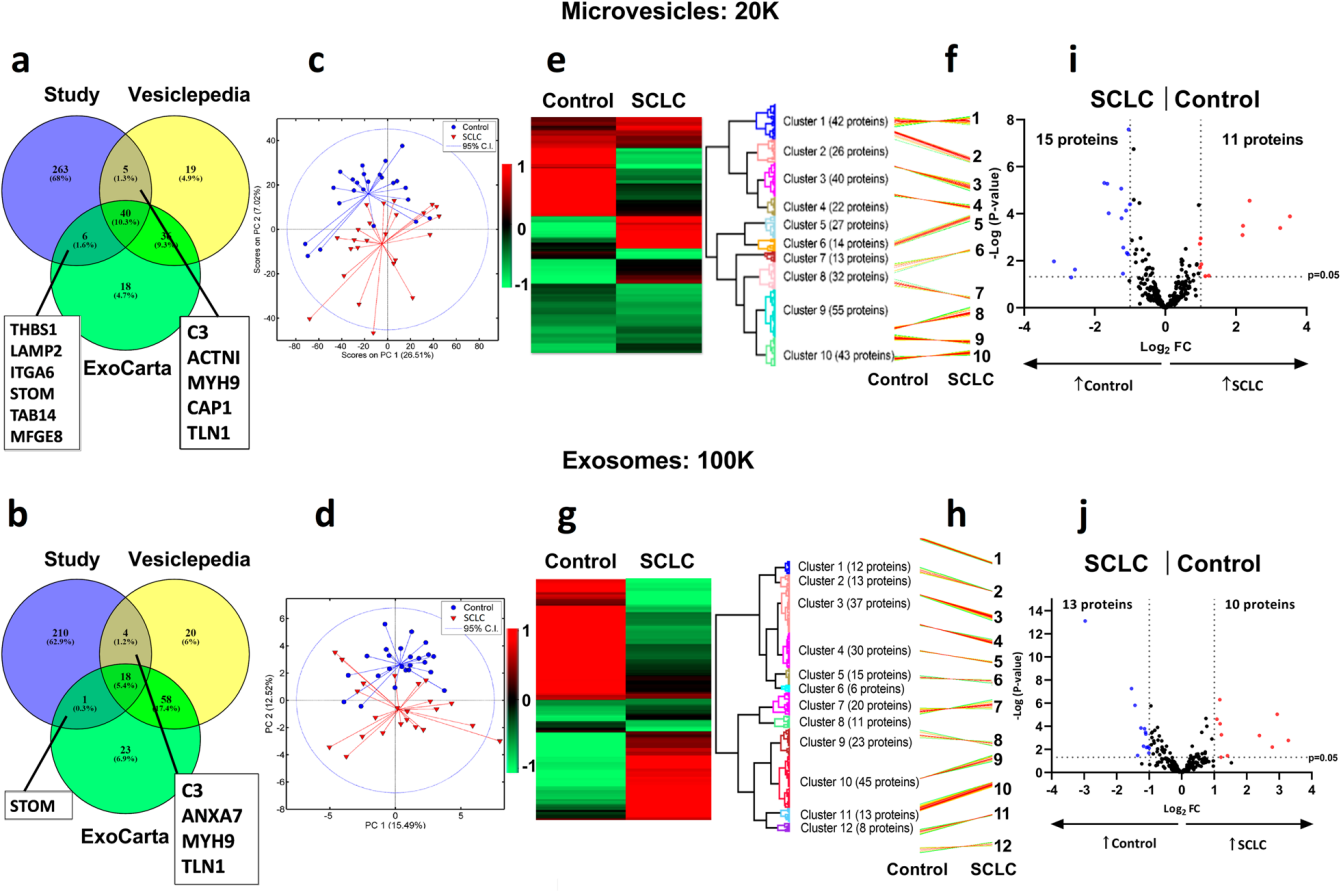
Proteomic analysis of circulating microvesicles and exosomes. **a** For the MV samples, a total of 51 proteins overlapped with the top 100 proteins from at least one of the EV databases, Vesiclepedia and ExoCarta (Additional file 2: Table S1) with 40 proteins common to all three groups and six and five proteins being shared between the study and ExoCarta and Vesiclepedia, respectively. **b** Of the 233 identified proteins in exosomes, 23 overlap with the top 100 EVs from at least one of the EV databases, of which 18 proteins were common to all three groups and one and four proteins are shared between the study and ExoCarta and Vesiclepedia, respectively. PCA revealed a clear separation between Controls (blue circles) and SCLC patients (Baseline, red triangles) along the second principal component for 20 K (**c**) and 100 K (**d**). Hierarchical clustering analysis revealed 10 distinct protein clusters, a heatmap (**e**) and their respective profile plots (**f**) for the MV samples, and 12 distinct protein clusters, a heatmap (**g**) and profile plots (**h**) for the exosome samples. The heatmaps depict LFQ-values normalized to Z-score, while the profile plots depict the expression patterns of proteins clustered in each cluster. To investigate potential diagnostic markers for both EV-samples, volcano plots depicting upregulated proteins for SCLC (red) versus controls (blue) were prepared according to fold change (Log_2_ FC ≥ 1 or ≤ − 1) and p-value = 0.05 (grey dotted lines). **i** For the 20 K sample, 11 proteins were significantly upregulated in the SCLC and 15 proteins in the control group. **j** For the 100 K sample, 10 proteins were significantly upregulated in the SCLC and 13 proteins in the control group. *SCLC* small cell lung cancer, *MV* microvesicle, *EVs* extracellular vesicles, *PCA* principle component analysis, *PC* principal component, *CI* confidence interval, *LFQ* label-free quantification, *FC* fold change

Plasma proteins of circulating MVs and exosomes were characterized and confirmed as previously described [26] and in accordance with the Minimal Information for Studies of Extracellular Vesicles (MISEV) criteria [27]. Due to analytical troubleshooting, only 23 of the 24 SCLC samples could be used to investigate exosomes. In total, 314 proteins were identified in MVs and 233 proteins in exosomes. For MVs, 51 of the identified proteins accorded with the top 100 EV proteins from either Vesiclepedia or ExoCarta; of these, 36 proteins corresponded to both databases (Fig. 2a; Additional file 2: Table S1). For the exosome samples, 18 proteins overlapped with the top 100 EV identified proteins from both Vesiclepedia and ExoCarta (Fig. 2b; Additional file 2: Table S1).

Patterns in data were visualized using PCA (Fig. 2c, d). Interestingly, samples cluster according to the health state of each individual along the first and the second principal components (PC1, PC2), indicating significant differences in MV (Fig. 2c) and exosome (Fig. 2d) protein profiles among HCs and SCLC patients.

For the MV samples (20 K), 10 distinct protein clusters were identified (Fig. 2e) with characteristic profiles (Fig. 2f). For the exosome samples (100 K), 12 distinct protein clusters were identified (Fig. 2g) with characteristic profiles (Fig. 2h). Additional information related to the distribution of proteins within clusters is summarized in Additional file 3: Table S2. Volcano plots illustrating the magnitude changes in protein expression between SCLC patients and HCs for 20 K and 100 K samples are depicted in Fig. 2i, j, respectively. Results from the functional enrichment analyses performed on the significantly up- and downregulated proteins are presented in Additional file 4: Table S3. For the 20 K samples, the proteins upregulated in SCLC patients are related to cell- and cell–matrix adhesion, integrin-mediated signaling, and extracellular matrix organization with an ES ≥ 3.18 (Additional file 4: Table S3). For the 100 K samples, the upregulated proteins are related to among others, complement activation, cytolysis, lipoprotein-associated processes, and cholesterol transport with an ES ≥ 4.56 (Additional file 4: Table S3). No GOBP terms were found to be enriched for the proteins downregulated in the 20 K samples. However, a significant association was observed between these downregulated proteins and biological processes, such as platelet degranulation, blood coagulation, hydrogen peroxide catabolic process, extracellular matrix disassembly and -organization, cellular protein metabolic processes, and oxygen- and lipid transport (Additional file 4: Table S3). For the 100 K samples, the proteins downregulated in SCLC patients are related to among others complement activation, proteolysis, receptor-mediated endocytosis, phagocytosis, and immune response with an ES ≥ 4.46 (Additional file 4: Table S3).

### Dynamics of microvesicle and exosomal proteins in SCLC diagnosis

Protein expression analysis revealed 62 proteins being differentially expressed between SCLC patients and HCs for the MV samples, where 26 proteins were upregulated and 36 were downregulated in SCLC patients (Additional file 5: Table S4). For the exosome samples, 68 proteins were differentially expressed, whereof 29 proteins were upregulated and 39 were downregulated in SCLC patients compared to HCs (*p* < 0.05) (Additional file 5: Table S4). A supplementary venn diagram was created to illustrate the proteins uniquely up- and downregulated for 20 K or 100 K, respectively, and those that are commonly expressed (Additional file 1: Fig. S1). Significantly differentially expressed proteins between SCLC patients and HCs were selected for additional analysis (*p* < 0.05 and Log_2_ FC ≥ 1 or ≤ -1) (Additional file 5: Table S4). For MV samples, 11 proteins were upregulated and 15 proteins downregulated in SCLC patients compared to HCs and fulfilled the FC criteria (Fig. 2i). For the exosome samples, 10 proteins were upregulated and 13 proteins downregulated in SCLC compared to HCs and fulfilled the FC criteria (Fig. 2j). Table 2 presents the 10 proteins common between MVs and exosomes with Log_2_ FC ≥ 1 or ≤ -1 in at least one of the vesicle types, the 16 proteins unique for MVs, and the 17 proteins unique for exosomes (data based on both on *p*-values < 0.05 and Log_2_ FC ≥ 1 or ≤ − 1).

**Table 2:** Significantly differentially expressed proteins for 20 K and 100 K comparing SCLC to the control group

SCLC|control: common proteins in microvesicle (20 K) and exosome (100 K) samples						
Uniprot ID	Gene name	Protein name	Log_2_ FC		*p*-value	
			20 K	100 K	20 K	100 K
P02741	CRP	C-reactive protein	3.5	1.2	0.0001	0.0016
P15144	ANPEP	Aminopeptidase N	3.2	2.4	0.0004	0.0006
P0DJI8	SAA1	Serum amyloid A-1 protein	2.4	2.9	< 0.0001	< 0.0001
P02763	ORM1	Alpha-1-acid glycoprotein 1	1.0	0.4	0.0011	0.0474
P02750	LRG1	Leucine-rich alpha-2-glycoprotein	0.9	1.2	0.0140	< 0.0001
P00738	HP	Haptoglobin	0.9	1.2	0.0004	< 0.0001
P06396	GSN	Gelsolin	− 1.0	− 0.7	< 0.0001	0.0001
P69905	HBA1	Hemoglobin subunit alpha	− 1.2	− 1.4	0.0002	< 0.0001
P06727	APOA4	Apolipoprotein A-IV	− 1.1	− 0.6	0.0001	0.0109
P68871	HBB	Hemoglobin subunit beta	− 1.6	− 0.9	< 0.0001	0.0003

SCLC|control: proteins detected only in the microvesicle samples (20 K)				
Uniprot ID	Gene name	Protein name	Log_2_ FC	*p*-value
P02786	TFRC	Transferrin receptor protein 1	2.2	0.0003
Q08380	LGALS3BP	Galectin-3-binding protein	2.2	0.0008
P05164	MPO	Myeloperoxidase	1.2	0.0424
Q13418	ILK	Integrin-linked protein kinase	1.0	0.0140
P23229	ITGA6	Integrin alpha-6	1.0	0.0193
Q96PD5	PGLYRP2	*N*-acetylmuramoyl-l-alanine amidase	− 1.0	< 0.0001
O00391	QSOX1	Sulfhydryl oxidase 1	− 1.1	0.0052
P02724	GYPA	Glycophorin-A	− 1.1	0.0046
P00915	CA1	Carbonic anhydrase 1	− 1.2	0.0028
P32119	PRDX2	Peroxiredoxin-2	− 1.2	0.0351
Q15582	TGFBI	Transforming growth factor-beta-induced protein ig-h3	− 1.2	< 0.0001
P02730	SLC4A1	Band 3 anion transport protein	− 1.6	0.0001
P02042	HBD	Hemoglobin subunit delta	− 1.7	< 0.0001
P16157	ANK1	Ankyrin-1	− 2.6	0.0233
P11277	SPTB	Spectrin beta chain erythrocytic	− 2.7	0.0502
P02549	SPTA1	Spectrin alpha chain erythrocytic 1	− 3.2	0.0106

SCLC|control: proteins detected only in the exosome samples (100 K)				
Uniprot ID	Gene name	Protein name	Log_2_ FC	p-value
P0DJI8	SAA2	Serum amyloid A-1 protein	3.3	0.0016
P02655	APOC2	Apolipoprotein C-II	2.8	0.0062
P08519	LPA	Apolipoprotein(a)	1.4	0.0346
Q92496	CFHR4	Complement factor H-related protein 4	1.2	0.0005
P04114	APOB	Apolipoprotein B	1.1	< 0.0001
P00736	C1R	Complement C1r subcomponent	− 1.0	0.0077
Q06830	PRDX1	Peroxiredoxin-1	− 1.0	0.0203
P05160	F13B	Coagulation factor XIII B chain	− 1.0	0.0060
P48740	MASP1	Mannan-binding lectin serine protease 1	− 1.1	0.0067
P02745	C1QA	Complement C1q subcomponent subunit A	− 1.1	0.0005
P00488	F13A1	Coagulation factor XIII A chain	− 1.1	0.0003
P00739	HPR	Haptoglobin-related protein	− 1.1	0.0002
Q8WWZ8	OIT3	Oncoprotein-induced transcript 3 protein	– 1.2	0.0052
P03951	F11	Coagulation factor XI	−1.3	0.0001
Q9Y6R7	FCGBP	IgGFc-binding protein	−1.4	0.0333
Q15485	FCN2	Ficolin-2	−1.5	< 0.0001
P06312	IGKV4-1	Ig kappa chain V–IV region	– 3.0	< 0.0001

A Log_2_ FC ± 1 indicates a twofold increase (+) or decrease (−) in SCLC compared to controls.

*SCLC* small cell lung cancer, *FC* fold change

**Fig. 3a:**
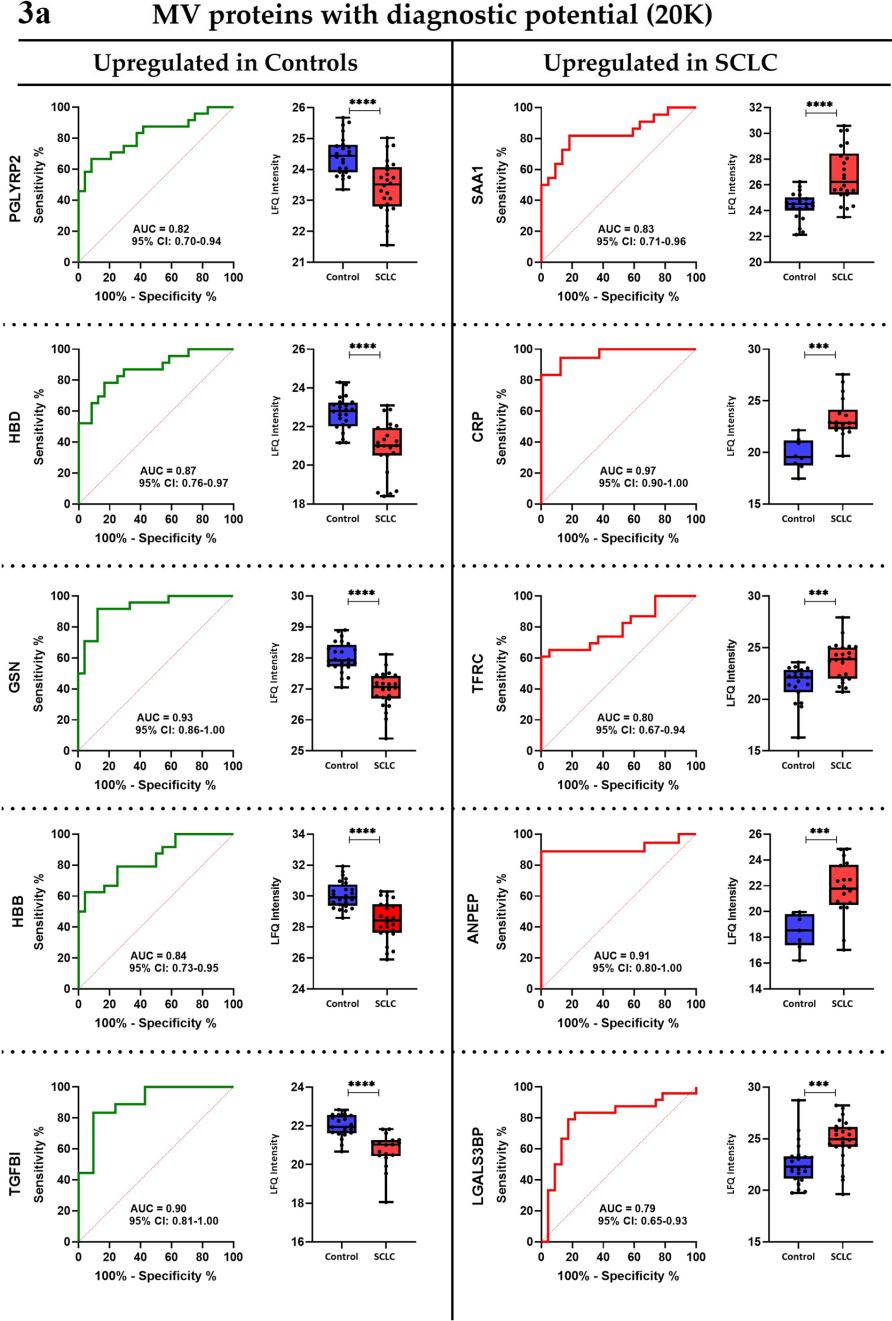

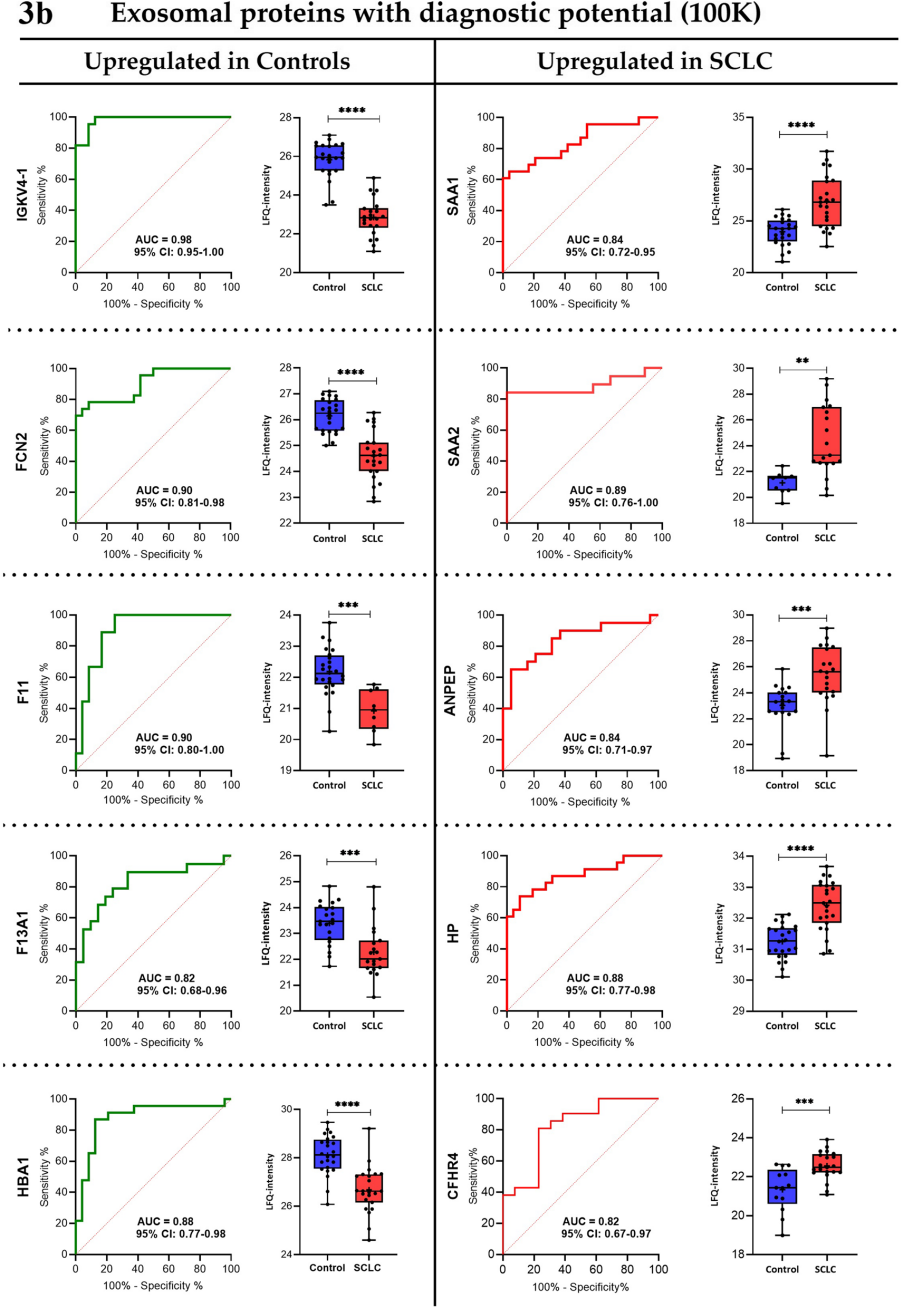
Receiver operating characteristic curves and boxplots of protein candidates for the 20 K samples. Proteins with diagnostic potential found to be upregulated in the SCLC patients were Serum amyloid A-1 protein (SAA1), C-reactive protein (CRP), Transferrin receptor protein 1 (TFRC), Aminopeptidase N (ANPEP), and Galectin-3-binding protein (LGALS3BP), while the proteins upregulated in the control group were Gelsolin (GSN), Transforming growth factor-beta-induced protein ig-h3 (TGFBI), Hemoglobin subunit beta and delta (HBB and HBD), and N-acetylmuramoyl-l-alanine amidase (PGLYRP2). Boxplots show non-logarithmic label-free quantification (LFQ) intensities excluding NaN (missing) values. *AUC* area under the curve, *CI* confidence interval, *SCLC* small cell lung cancer, *LFQ* label-free quantification. **b **Receiver operating characteristic curves and boxplots of protein candidates for the 100 K samples. Proteins with diagnostic potential found to be upregulated in the SCLC patients were Serum amyloid A-1 and A-2 protein (SAA1 and SAA2), Aminopeptidase N (ANPEP), Haptoglobin (HP), and Complement factor H-related protein 4 (CFHR4), and the proteins upregulated in the control group were Ig kappa chain V–IV region (IGKV4-1), Ficolin-2 (FCN2), Coagulation factor XI (F11), Coagulation factor XIII A chain (F13A1), and Hemoglobin subunit alpha (HBA1). Boxplots show non-logarithmic label-free quantification (LFQ) intensities and exclude NaN (missing) values. *AUC* area under the curve, *CI* confidence interval, *SCLC* small cell lung cancer, *LFQ* label-free quantification

To assess the diagnostic capacity of the most significantly expressed proteins in the groups, receiver operating characteristics (ROC) analysis was conducted. Top 10 proteins (with AUC ≥ 0.8) for the MV (20 K) and exosome (100 K) samples, respectively, are visualized in Fig. 3a, b, and additional information can be found in Additional file 6: Table S5.

In addition to the top 10 most distinct proteins among groups, a range of proteins which have previously been found in association with cancer also revealed acceptable sensitivity and specificity (Table 3).

**Table 3:** Potential cancer-related EV biomarkers for SCLC diagnosis based on ROC analysis

20 K SCLC|control						
Protein	AUC	95% CI	p-value	Sensitivity (%)	Specificity (%)	Log2 FC
ILK	0.76	0.55–0.87	0.0192	75	59	1.0
ORM1	0.76	0.62–0.89	0.0021	79	54	1.0
GYPA	0.75	0.59–0.90	0.0092	77	64	1.0
QSOX1	0.79	0.63–0.94	0.0047	87	63	− 1.1
CA1	0.80	0.65–0.94	0.0011	83	74	− 1.2
PRDX2	0.73	0.58–0.88	0.0083	77	67	− 1.2
ANK1	0.76	0.55–0.96	0.0301	78	70	− 2.6
ITGA6	0.74	0.59–0.90	0.0084	59	83	− 2.6
SPTB	0.75	0.54–0.96	0.0419	63	80	− 2.7
SPTA1	0.81	0.65–0.98	0.0046	82	76	− 3.2

100 K SCLC|control						
Protein	AUC	95% CI	*p*-value	Sensitivity (%)	Specificity (%)	Log_2_ FC
APOC2	0.81	0.65–1.0	0.0140	78	89	2.8
LRG1	0.84	0.72–0.96	0.0002	82	75	1.2
APOB	0.86	0.76–0.96	< 0.0001	83	75	1.1
PRDX1	0.74	0.53–0.86	0.0407	89	50	− 1.0
OIT3	0.74	0.59–0.83	0.0058	76	65	− 1.2

A Log_2_ FC ± 1 indicates a twofold increase (+) or decrease (−) in SCLC compared to controls.

*SCLC* small cell lung cancer, *AUC* area under the curve, *CI* confidence interval, *FC* fold change, *CA1* carbonic anhydrase 1, *QSOX1* sulfhydryl oxidase 1, *ILK* integrin-linked protein kinase, *ORM1* alpha-1-acid glycoprotein 1, *ANK1* ankyrin-1, *GYPA* glycophorin-A, *ITGA6* integrin alpha-2, *PRDX2* peroxiredoxin-2, *SPTB* spectrin beta chain erythrocytic, *SPTA1* Spectrin alpha chain erythrocytic 1, *APOC2* Apolipoprotein C-II, *LRG1* leucine-rich alpha-2-glycoprotein, *APOB* apolipoprotein B, *PRDX1* peroxiredoxin-1, *OIT3* oncoprotein-induced transcript 3 protein

## Discussion

Small cell lung cancer is the most aggressive form of lung cancer with early metastasis resulting in poor prognosis. Therefore, it would be favourable to identify characteristic markers to improve the early detection of SCLC. We present results of a comprehensive untargeted quantitative MS-based proteomics analysis on plasma-derived MVs and exosomes from HCs and newly diagnosed SCLC patients, aiming at identifying easily accessible putative markers.

In our study, 233 exosomal and 314 MV-derived proteins were investigated for diagnostic potential in SCLC. We observed several tumor-derived MV and exosomal proteins capable of differentiating between SCLC patients and HCs with high efficacy (Fig. 3a, b; Table 3). Uniquely for the MV samples, the upregulated proteins were found to be related to cell adhesion, integrin-mediated signaling, and extracellular matrix organization, while the upregulated exosomal proteins are exclusively related to cytolysis, and lipid- and cholesterol remodelling. Moreover, for the MV samples, the downregulated proteins were found to be uniquely related to platelet degranulation, blood coagulation, hydrogen peroxide catabolic process, and oxygen- and bicarbonate transport, whereas the downregulated exosomal proteins are specifically related to receptor-mediated endocytosis, proteolysis, immune response, and phagocytosis (Additional file 4: Table S3). Interestingly, we found that complement activation and -regulation is associated with both the up- and downregulated exosomal proteins, indicating an important role of the complement cascade in SCLC pathogenesis (Additional file 4: Table S3). Despite these differences in the biological pathways associated with MV-derived and exosomal proteins, the proteome manifestation of MVs and exosomes for SCLC diagnosis appears to be partly comparable according to Fig. 3a, b; Additional file 4: Table S3). In the following, we attempt to syndicate markedly expressed proteins that are shared in SCLC, NSCLC, and other cancer types, and unraveling those that are novel for SCLC.

Chronic inflammation is a key promoter of carcinogenesis and its acceleration in cancer patients is linked to disease progression [28]. For SCLC patients, we observed both an upregulation (i.e. CRP, TFRC, ANPEP, SAA1, SAA2, ORM1, and HP) and downregulation (i.e. FCN2) of inflammation markers. Similar findings have previously been described in lung cancer patients [29–35]. Moreover, we also observed a significantly upregulated expression of proteins related to tumorigenesis, metastasis, and cell proliferation (ILK, ITGA6, LGALS3BP, and LRG1) in SCLC patients compared to HCs, and similar findings have also been documented for NSCLC patients [36–39]. Additionally, the two tumor-metastatic markers, ANK1 and GYPA, were also identified as downregulated in SCLC patients. These findings were also confirmed previously in NSCLC patients [40, 41]. Importantly, we observed a ninefold decrease in MV-derived α-and β subunits of spectrins, indicating that SCLC microvesicles may be involved in cell adhesion, cell spreading, and metastasis. Comparable aberrant decreases of spectrin subunits were also identified in primary tumors and body fluids from patients with NSCLC and other cancer types [40, 42]. The downregulation of the tumor suppressor marker, GSN, detected in our study has also been reported for NSCLC [43]. Another protein involved in tumourigenesis and identified as significantly diminished in SCLC in our study population was CA1. Similarly, decreased CA1 protein expression has been observed in NSCLC patients [44]. However, in contrast, also augmented levels of CA1 in serum have been observed in early stage NSCLC patients and in tumor tissues from SCLC patients [45, 46]. Furthermore, the downregulated expression of the oncoprotein, OIT3, the immunomodulatory protein, PGLYRP2, and the blood coagulation factor X1 (F11) have shown high diagnostic ability to distinguish between SCLC patients and HCs. Parallel findings have also been recognized for other cancer types [47–49] but not in NSCLC.

In the current SCLC cohort, downregulation of the inflammation marker (IGKV4-1), the tumor aggressivity associated marker (QSOX1), and the tumor suppressor marker (TGFβ1) were observed. Interestingly, these proteins have been reported to be upregulated in NSCLC and other solid tumors [50–53]. Hence, upon validation, we believe that measurements of all three proteins may have potentials in improving SCLC diagnosis.

Additionally, we observed downregulation of blood hemoglobin markers (HBA1, HBB, and HBD) and peroxiredoxins (PRDX1 and PRDX2) in patients with SCLC, which is opposite to the upregulated levels previously observed in lung cancer patients, predominantly in NSCLC patients [54, 55], except for PRDX2 which has been reported to be downregulated in NSCLC [56]. Recently, it has been reported that decreased hemoglobin‐to‐red blood cell distribution width ratio in NSCLC and SCLC patients is associated with poor prognosis, which is suggested to be caused by an increased amount of hypoxic cells, contributing to an aggressive tumor phenotype [57]. This is in agreement with our data, suggesting that oxidative stress may be a driver in or a consequence of SCLC pathogenesis. Furthermore, SCLC patients exhibited increased protein expressions of lipid transport markers (APOB and APOC2), but decreased levels of APOA4 (Additional file 5: Table S4) when compared to HCs. Previously, APOB has been shown to be downregulated in NSCLC patients [58], thus revealing the ability of APOB to discriminate between NSCLC and SCLC. Remarkably, APOC3 protein expression has been previously shown to be significantly lower in SCLC tissues compared to both NSCLC and normal tissue [59]. However, these results may be influenced by the effect of non-fasting patients at time of diagnosis in our study and probable contamination of lipoproteins in the EV fractions. Therefore, further research should be conducted to confirm our findings.

The significant downregulation of coagulation factor XIII A chain (F13A1) and upregulation of the complement factor H-related protein 4 (CFHR4) in SCLC compared to HCs has not yet been identified in other cancers, including lung cancer. In the study we present evidence that these markers could serve as future diagnostic markers in SCLC with an AUC of 0.82 for F13A1 and CFHR4 (95% CI: 0.69–0.96 and 95% CI: 0.67–0.97, respectively). Cancer patients are generally hypercoagulable, and hence, associated with a high risk of venous thromboembolism [60]. Therefore, the downregulation of F13A1 in SCLC is surprising, but may indicate a novel tumor suppressing role of blood coagulation in SCLC pathogenesis, which is supported by the similar downregulated expression of F11 in SCLC patients in the current study.

CFHR4, a soluble regulator of the complement cascade, is generally known to boost complement activation [61], a process presumed to contribute to tumor growth [62]. The upregulation of CFHR4 observed in SCLC patients may suggest that complement activation plays a role in SCLC pathogenesis. However, previous studies have reported a significant downregulation of membrane-bound complement regulators (CD46, CD55, and CD59) in SCLC compared to other cancers, including NSCLC [63]. Thus, our finding indicates that soluble CFHR4 may be specifically expressed in SCLC as a positive regulator of complement activation.

The present study holds some limitations regarding small sample size, EV isolation, and methodological aspects of data analyses. Even though the small number of patients may bias the results, we identified several proteins that showed marked differences in their expression levels among SCLC patients versus HCs. The reduced patient size and the limited number of patients with early stage disease (n = 1) restricts possible correlations between the early and advanced stages. Additional studies including more early stage patients would be ideal in order to answer this problematic. Other confounding factors possibly impacting our results include co-morbidity and cachexia. However, the last mentioned is rarely the case in patients considered suitable for chemotherapy. Regarding methodology, the MS-datasets contain many missing values, which could result in loss of some potentially important comparisons. However, whether the missing values are a result of LFQ-intensities below the detection limit, or whether the protein is simply not expressed in that particular patient, is uncertain. The number of missing values could probably have been reduced if we used another experimental design. In the present study, we have used the data dependent aquisition (DDA) approach. However, in the last couple of decades another principle of analysis, data independent acquisition (DIA), has emerged that possesses some advantages. DIA has a limited number of missing values compared with DDA due to the stochastic sampling of the latter [64] and DIA may have a higher sensitivity although a direct comparison between the methods is still missing [65]. However, one of the major limitations with DIA is the need to generate spectral libraries for data processing [65]. This requires much higher sample amounts than the limited sample amount present for this investigation. Due to this limitation we have therefore used the well established DDA technique and apparently, the two approaches typically quantify a similar number of peptides and proteins in a single shot analysis [65, 66]. It has been reported by Cox et al. [67] that using the match between runs option in the MaxQuant analysis is another way to reduce the number of missing values. If match between runs were not used, we would probably loss some of the significant protein markers. Therefore, in our study, we used match between runs to allow for the identification of more proteins. The use of EVs as a source of biomarkers should also be noted in this section, as plasma proteins may adhere to EVs and therefore not be cargo in the EVs. However, that may not exclude these proteins as possible diagnostic biomarkers. The stringency of data filtration is subjective and with harsh filtration techniques, the risk of oversight of important markers cannot be excluded. However, without filtrations, the risk of introducing contaminants into the dataset is plausible, leading to the risk of biased results. Moreover, this study has solely compared SCLC patients with HCs. The diagnostic efficiency may be lower when compared to other cancer patients, e.g. regarding inflammatory markers that are generally upregulated in cancer patients. Lastly, for future studies, having focus on glycosylation could improve the identification of biomarkers in SCLC. Thus, since glycosylation may be altered in diseases such as cancer it will be an advantage in future studies to examine the role of glycolysis in SCLC using glycoproteomics.

## Conclusions

To our knowledge, this is the first study to identify single proteins (CFHR4 and F13A1) and a panel of proteins as potential candidates for SCLC diagnosis using an untargeted quantitative proteomic approach. We observed an altered expression of proteins related to inflammation, coagulation, complement activation, hematological dysfunction, lipid metabolism, and hydrogen peroxide catabolism, as opposed to expression patterns observed in NSCLC and other cancers. However, validation studies verifying these proteins as candidate markers in SCLC are warranted.

## Supplementary Information

**Additional file 1: Fig. S1.** A supplementary Venn diagram was created to illustrate the proteins uniquely up- and downregulated for 20 K or 100 K, respectively, and those that are commonly expressed. Venn diagram describing differences between MV and exosomal proteins.

**Additional file 2: Table S1.** Top 100 proteins related to EVs from EV databases ExoCarta and Vesiclepedia.

**Additional file 3: Table S2.** Protein clusters and gene names.

**Additional file 4: Table S3.** Functional Enrichment analysis.

**Additional file 5: Table S4.** Significantly regulated proteins in 20 K and 100 K samples.

**Additional file 6: Table S5**. Potential diagnostic proteins.

## Data Availability

The 20 K and 100 K MS raw data for this manuscript has been uploaded in ProteomeXchange Consortium via the PRIDE partner repository with the dataset identifier PXD028944 and PXD028885, respectively.

## Abbreviations

SCLC: Small cell lung cancer
NSCLC: Non-small cell lung cancer
CT: Computed tomography
FNA: Fine-needle aspiration
EVs: Extracellular vesicles
MVs: Microvesicles
MS: Mass spectrometry
TNM: Tumor, lymph node, metastasis
HCs: Healthy controls
LFQ: Label-free quantification
MISEV: Minimal Information for Studies of Extracellular Vesicles
SD: Standard deviations
PCA: Principal component analysis
FC: Fold change
GOBP: Gene ontology biological process
DAVID: The Database for Annotation, Visualization, and Integrated Discovery
PC: Principal components
ES: Enrichment score
ROC: Receiver operating characteristics
AUC: Area under the curve
CI: Confidence interval
