# Supplementary figures and images for: Circulating microvesicles and exosomes in small cell lung cancer by quantitative proteomics
Source: Clin Proteomics. 2022 Jan 7;19:2. doi: 10.1186/s12014-021-09339-5 (PMC8903681; doi:10.1186/s12014-021-09339-5)

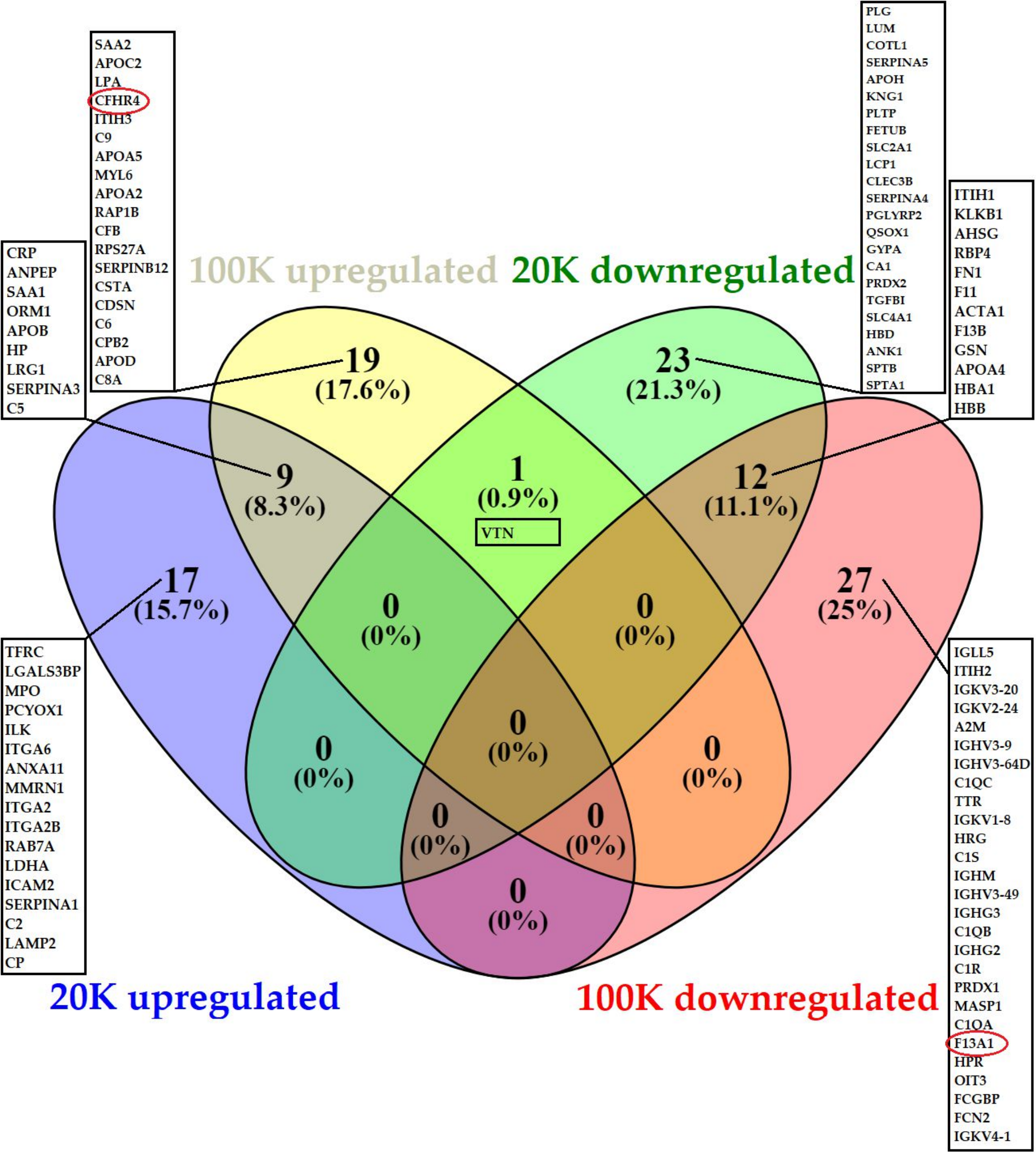

Supplement: Supplementary file 1 — Additional file 1: Fig. S1. A supplementary Venn diagram was created to illustrate the proteins uniquely up- and downregulated for 20 K or 100 K, respectively, and those that are commonly expressed. Venn diagram describing differences between MV and exosomal proteins. [file 12014_2021_9339_MOESM1_ESM.pdf]
