# Supplementary material for: Circulating microvesicles and exosomes in small cell lung cancer by quantitative proteomics
Source: Clin Proteomics. 2022 Jan 7;19:2. doi: 10.1186/s12014-021-09339-5 (PMC8903681; doi:10.1186/s12014-021-09339-5)
Supplement: Supplementary file 2 — Additional file 2: Table S1. Top 100 proteins related to EVs from EV databases ExoCarta and Vesiclepedia. [file 12014_2021_9339_MOESM2_ESM.pdf]

Table S1. Top 100 proteins related to EVs from EV databases ExoCarta and Vesiclepedia

	**20K = 314 identified proteins**		
	**Protein IDs**	**Protein names**	**T: Gene names**
1	A0A0A0MS15	Immunoglobulin heavy variable 3-49	IGHV3-49
2	A0A0B4J1V2	Immunoglobulin heavy variable 2-26	IGHV2-26
3	A0A0C4DH33	Immunoglobulin heavy variable 1-24	IGHV1-24
4	A0A0C4DH38	Immunoglobulin heavy variable 5-51	IGHV5-51
5	O00194;P51159-2;P51159	Ras-related protein Rab-27B	RAB27B
6	O00299	Chloride intracellular channel protei	CLIC1
7	O00391-2;O00391	Sulfhydryl oxidase 1	QSOX1
8	O14791-2;O14791;O14791-3	Apolipoprotein L1	APOL1
9	P19105;O14950	Myosin regulatory light chain 12A;M	MYL12A;MYL12B
10	O43707;O43707-2;O43707-3	Alpha-actinin-4	ACTN4
11	O43866	CD5 antigen-like	CD5L
12	O75083;O75083-3	WD repeat-containing protein 1	WDR1
13	O75131;Q86YQ8-2;Q9HCH3-2	Copine-3;Copine-8;Copine-5;Copine-	CPNE3;CPNE8;CPNE5;CPNE
14	O75390	Citrate synthase, mitochondrial	CS
15	O75636-2;O75636	Ficolin-3	FCN3
16	O75882-3;O75882-2;O75882	Attractin	ATRN
17	O95445;O95445-2	Apolipoprotein M	APOM
18	O95810	Serum deprivation-response protein	SDPR
19	O95866-4;O95866-5;O95866-	Protein G6b	G6B
20	P00338-5;P00338-2;P00338;P	L-lactate dehydrogenase A chain	LDHA
21	P00387-2;P00387;P00387-3	NADH-cytochrome b5 reductase 3;N	CYB5R3
22	P00450;CON__ENSEMBL:ENSI	Ceruloplasmin	CP
23	P00488	Coagulation factor XIII A chain	F13A1
24	P00491	Purine nucleoside phosphorylase	PNP
25	P00558-2;P00558	Phosphoglycerate kinase 1	PGK1
26	P00734;CON__P00735	Prothrombin;Activation peptide frag	F2
27	P00736	Complement C1r subcomponent;Coi	C1R
28	P00738;P00738-2	Haptoglobin;Haptoglobin alpha chai	HP
29	P00739;P00739-2	Haptoglobin-related protein	HPR
30	P00742	Coagulation factor X;Factor X light cl	F10
31	P00747;Q15195	Plasminogen;Plasmin heavy chain A;	PLG
32	P00748	Coagulation factor XII;Coagulation fa	F12
33	P00751;P00751-2	Complement factor B;Complement f	CFB
34	P00915	Carbonic anhydrase 1	CA1
35	P01008	Antithrombin-III	SERPINC1
36	P01009;P01009-2;P01009-3	Alpha-1-antitrypsin;Short peptide frc	SERPINA1
37	P01011;P01011-2;P01011-3	Alpha-1-antichymotrypsin;Alpha-1-a	SERPINA3
38	P01019	Angiotensinogen;Angiotensin-1;Angi	AGT
39	P01023	Alpha-2-macroglobulin	A2M
40	P01024	Complement C3;Complement C3 bet	C3
41	P01031;CON__Q1A7A4	Complement C5;Complement C5 bet	C5
42	P01040	Cystatin-A;Cystatin-A, N-terminally p	CSTA
43	P01042;P01042-2;P01042-3	Kininogen-1;Kininogen-1 heavy chair	KNG1
44	P01591	Immunoglobulin J chain	IGJ
45	P01594;P01593	Ig kappa chain V-I region AU;Ig kapp	IGKV1-33;IGKV1D-33

46	P01619	Ig kappa chain V-III region B6	IGKV3-20
47	P01743	Ig heavy chain V-I region HG3	IGHV1-46
48	P01764	Ig heavy chain V-III region 23	IGHV3-23
49	P01782;P0DP04	Ig heavy chain V-III region DOB	IGHV3-9;IGHV3-43D
50	P01833	Polymeric immunoglobulin receptor;	PIGR
51	P01834	Ig kappa chain C region	IGKC
52	P0DOX5;P01857	Ig gamma-1 chain C region	IGHG1
53	P01859	Ig gamma-2 chain C region	IGHG2
54	P01860	Ig gamma-3 chain C region	IGHG3
55	P01861	Ig gamma-4 chain C region	IGHG4
56	P01871;P01871-2	Ig mu chain C region	IGHM
57	P01876	Ig alpha-1 chain C region	IGHA1
58	P01889;P13747;P17693-6;P17	HLA class I histocompatibility antiger	HLA-B
59	P01903;P01906	HLA class II histocompatibility antige	HLA-DRA
60	P02042;CON__Q3SX09;P0210	Hemoglobin subunit delta	HBD
61	P02549-2;P02549	Spectrin alpha chain, erythrocytic 1	SPTA1
62	P02647	Apolipoprotein A-I;Proapolipoprotei	APOA1
63	P02649;CON__Q03247	Apolipoprotein E	APOE
64	P02654	Apolipoprotein C-I;Truncated apolip	APOC1
65	P02656	Apolipoprotein C-III	APOC3
66	P02671;P02671-2;CON__P026	Fibrinogen alpha chain;Fibrinopeptic	FGA
67	P02675;CON__P02676	Fibrinogen beta chain;Fibrinopeptide	FGB
68	P02679-2;P02679	Fibrinogen gamma chain	FGG
69	P02724-3;P02724-2;P02724	Glycophorin-A	GYPA
70	P02730;P02730-2	Band 3 anion transport protein	SLC4A1
71	P02741;P02741-2	C-reactive protein;C-reactive protein	CRP
72	P02745	Complement C1q subcomponent sul	C1QA
73	P02746	Complement C1q subcomponent sul	C1QB
74	P02748;CON__Q3MHN2	Complement component C9;Comple	C9
75	P02749;CON__P17690	Beta-2-glycoprotein 1	APOH
76	P02750	Leucine-rich alpha-2-glycoprotein	LRG1
77	P02751-3;P02751-1;P02751-1	Fibronectin;Anastellin;Ugl-Y1;Ugl-Y2	FN1
78	P02753	Retinol-binding protein 4;Plasma ret	RBP4
79	P02760	Protein AMBP;Alpha-1-microglobulir	AMBP
80	P02763	Alpha-1-acid glycoprotein 1	ORM1
81	P02765	Alpha-2-HS-glycoprotein;Alpha-2-HS	AHSG
82	P02766;Q5U7I5	Transthyretin	TTR
83	P02774;P02774-3;P02774-2;C	Vitamin D-binding protein	GC
84	P02776	Platelet factor 4;Platelet factor 4, sh	PF4
85	P02786	Transferrin receptor protein 1;Trans	TFRC
86	P02787	Serotransferrin	TF
87	P02788-2;P02788	Lactotransferrin;Lactoferricin-H;Kalic	LTF
88	P02790	Hemopexin	HPX
89	P02792	Ferritin light chain	FTL
90	P03951-2;P03951	Coagulation factor XI;Coagulation fa	F11
91	P03952	Plasma kallikrein;Plasma kallikrein h	KLKB1
92	P04003	C4b-binding protein alpha chain	C4BPA
93	P04004;CON__Q3ZBS7	Vitronectin;Vitronectin V65 subunit;	VTN
94	P04040	Catalase	CAT
95	P04075;P04075-2	Fructose-bisphosphate aldolase A	ALDOA

96	P04083	Annexin A1	ANXA1
97	P04114	Apolipoprotein B-100;Apolipoprotei	APOB
98	P04180	Phosphatidylcholine-sterol acyltrans	LCAT
99	P04196	Histidine-rich glycoprotein	HRG
100	P04217;CON__Q2KJF1	Alpha-1B-glycoprotein	A1BG
101	P04275;P04275-2	von Willebrand factor;von Willebran	VWF
102	P04278;P04278-4;P04278-2;P	Sex hormone-binding globulin	SHBG
103	P04406-2;P04406	Glyceraldehyde-3-phosphate dehydr	GAPDH
104	P04439;P04439-2	HLA class I histocompatibility antigen	HLA-A
105	P04792	Heat shock protein beta-1	HSPB1
106	P04899;P04899-4;P04899-6;P	Guanine nucleotide-binding protein	GNAI2
107	P05023-3;P05023-4;P05023;P	Sodium/potassium-transporting ATP	ATP1A1
108	P05089-2;P05089;P05089-3	Arginase-1	ARG1
109	P05106-2;P05106-3;P05106	Integrin beta-3	ITGB3
110	P05107	Integrin beta-2	ITGB2
111	P05141	ADP/ATP translocase 2;ADP/ATP tra	SLC25A5
112	P05154	Plasma serine protease inhibitor	SERPINA5
113	P05155-2;P05155;P05155-3;C	Plasma protease C1 inhibitor	SERPING1
114	P05156;CON__Q32PI4	Complement factor I;Complement fa	CFI
115	P05160	Coagulation factor XIII B chain	F13B
116	P05164-2;P05164;P05164-3	Myeloperoxidase;Myeloperoxidase;	MPO
117	P05362	Intercellular adhesion molecule 1	ICAM1
118	P05452;CON__Q2KIS7	Tetranectin	CLEC3B
119	P05543;CON__Q9TT36	Thyroxine-binding globulin	SERPINA7
120	P05546	Heparin cofactor 2	SERPIND1
121	P05556-2;P05556;P05556-5;P	Integrin beta-1	ITGB1
122	P06276	Cholinesterase	BCHE
123	P06396-2;P06396-4;P06396-3	Gelsolin	GSN
124	P06576	ATP synthase subunit beta, mitochon	ATP5B
125	P06681;P06681-3;P06681-2	Complement C2;Complement C2b fr	C2
126	P06702	Protein S100-A9	S100A9
127	P06727	Apolipoprotein A-IV	APOA4
128	P06733;P06733-2;P09104-2	Alpha-enolase	ENO1
129	P07225;CON__P07224	Vitamin K-dependent protein S	PROS1
130	P07339	Cathepsin D;Cathepsin D light chain;	CTSD
131	P07355-2;P07355;A6NMY6	Annexin A2;Putative annexin A2-like	ANXA2;ANXA2P2
132	P07357	Complement component C8 alpha cl	C8A
133	P07358	Complement component C8 beta ch	C8B
134	P07359	Platelet glycoprotein Ib alpha chain;	GP1BA
135	P07360	Complement component C8 gamma	C8G
136	P07384	Calpain-1 catalytic subunit	CAPN1
137	P07437;Q9BUF5;A6NNZ2	Tubulin beta chain	TUBB
138	P07737;CON__P02584	Profilin-1	PFN1
139	P07900;P07900-2;Q58FG0;Q1	Heat shock protein HSP 90-alpha	HSP90AA1
140	P07948-2;P07948	Tyrosine-protein kinase Lyn	LYN
141	P07996;P07996-2;P35442;P4	Thrombospondin-1	THBS1
142	P08133;P08133-2	Annexin A6	ANXA6
143	P08134;P61586;P62745	Rho-related GTP-binding protein Rho	RHOC;RHOA;RHOB
144	P08185	Corticosteroid-binding globulin	SERPINA6
145	P08514;P08514-2;P08514-3	Integrin alpha-IIb;Integrin alpha-IIb h	ITGA2B

146	P08519	Apolipoprotein(a)	LPA
147	P08567	Pleckstrin	PLEK
148	P08571	Monocyte differentiation antigen CD	CD14
149	P08575-4;P08575-9;P08575-1	Receptor-type tyrosine-protein phos	PTPRC
150	P08603;P08603-2;Q02985-2;(	Complement factor H	CFH
151	P08697;P08697-2;CON__P28{	Alpha-2-antiplasmin	SERPINF2
152	P08709-2;P08709	Coagulation factor VII;Factor VII light	F7
153	P09211	Glutathione S-transferase P	GSTP1
154	P09871	Complement C1s subcomponent;Co	C1S
155	P09972	Fructose-bisphosphate aldolase C	ALDOC
156	P0C0L4;P0C0L4-2;CON__ENSE	Complement C4-A;Complement C4 b	C4A
157	P0C0L5	Complement C4-B;Complement C4 b	C4B
158	P0DJI8;P0DJI9-2	Serum amyloid A-1 protein;Amyloid	SAA1
159	P0DMV9;P0DMV8;P0DMV8-2	Heat shock 70 kDa protein 1B;Heat s	HSPA1B;HSPA1A
160	P0DOX6		IGHM(2)
161	P0DOX7		IGKC(2)
162	P0DOY3;P0DOY2;P0CF74;A0M	Ig lambda-6 chain C region;Ig lambda	IGLC6;IGLC7
163	P0DP25;P0DP24;P0DP23		CALM3;CALM2;CALM1
164	P10321;P10321-2	HLA class I histocompatibility antiger	HLA-C
165	P10643	Complement component C7	C7
166	P10809	60 kDa heat shock protein, mitochor	HSPD1
167	P10909-4;P10909-6;P10909;P	Clusterin;Clusterin beta chain;Cluste	CLU
168	P11021	78 kDa glucose-regulated protein	HSPA5
169	P11142;P11142-2;P54652	Heat shock cognate 71 kDa protein	HSPA8
170	P11166	Solute carrier family 2, facilitated glu	SLC2A1
171	P11169;Q8TDB8-4;Q8TDB8-2;	Solute carrier family 2, facilitated glu	SLC2A3;SLC2A14
172	P11277;P11277-3;P11277-2;C	Spectrin beta chain, erythrocytic	SPTB
173	P11597;P11597-2	Cholesteryl ester transfer protein	CETP
174	P12259;CON__Q28107	Coagulation factor V;Coagulation fac	F5
175	P12273	Prolactin-inducible protein	PIP
176	P12814;P12814-2;P12814-3;P	Alpha-actinin-1	ACTN1
177	P13224;P13224-2	Platelet glycoprotein Ib beta chain	GP1BB
178	P13473;P13473-2;P13473-3	Lysosome-associated membrane gly	LAMP2
179	P13598	Intercellular adhesion molecule 2	ICAM2
180	P13671	Complement component C6	C6
181	P13796;P13796-2;Q14651	Plastin-2	LCP1
182	P14618;P14618-3;P14618-2;P	Pyruvate kinase PKM	PKM
183	P14770	Platelet glycoprotein IX	GP9
184	P14923;P35222	Junction plakoglobin	JUP
185	P15144	Aminopeptidase N	ANPEP
186	P15169;CON__Q2KJ83	Carboxypeptidase N catalytic chain	CPN1
187	P15924;P15924-2;P15924-3	Desmoplakin	DSP
188	P16157-14;P16157-8;P16157-	Ankyrin-1	ANK1
189	P16284-5;P16284-3;P16284-4	Platelet endothelial cell adhesion mc	PECAM1
190	P16671;P16671-4;P16671-3;P	Platelet glycoprotein 4	CD36
191	P17301	Integrin alpha-2	ITGA2
192	P18206;P18206-2;P18206-3	Vinculin	VCL
193	P18428	Lipopolysaccharide-binding protein	LBP
194	P19652	Alpha-1-acid glycoprotein 2	ORM2
195	P19823	Inter-alpha-trypsin inhibitor heavy cl	ITIH2

196	P19827;P19827-2;P19827-3;C	Inter-alpha-trypsin inhibitor heavy cl	ITIH1
197	P20701-2;P20701;P20701-3	Integrin alpha-L	ITGAL
198	P20742;P20742-2	Pregnancy zone protein	PZP
199	P20851-2;P20851	C4b-binding protein beta chain	C4BPB
200	P21333-2;P21333	Filamin-A	FLNA
201	P22352	Glutathione peroxidase 3	GPX3
202	P22792	Carboxypeptidase N subunit 2	CPN2
203	P23142;P23142-2;P23142-3;C	Fibulin-1	FBLN1
204	P23229-4;P23229-2;P23229-5	Integrin alpha-6;Integrin alpha-6 hea	ITGA6
205	P23528	Cofilin-1	CFL1
206	P25311	Zinc-alpha-2-glycoprotein	AZGP1
207	P25705-2;P25705;P25705-3	ATP synthase subunit alpha, mitochc	ATP5A1
208	P26038;P35241-3	Moesin	MSN
209	P27105;P27105-2	Erythrocyte band 7 integral membra	STOM
210	P27169	Serum paraoxonase/arylesterase 1	PON1
211	P27824-2;P27824;P27824-3	Calnexin	CANX
212	P29622	Kallistatin	SERPINA4
213	P30041	Peroxiredoxin-6	PRDX6
214	P30101	Protein disulfide-isomerase A3	PDIA3
215	P30740;P30740-2;P50452-2;C	Leukocyte elastase inhibitor	SERPINB1
216	P31151;Q86SG5	Protein S100-A7;Protein S100-A7A	S100A7;S100A7A
217	P31944	Caspase-14;Caspase-14 subunit p17,	CASP14
218	P32119	Peroxiredoxin-2	PRDX2
219	P35542	Serum amyloid A-4 protein	SAA4
220	P35579;P35579-2;P35749-4;P	Myosin-9	MYH9
221	P35858;P35858-2	Insulin-like growth factor-binding pro	IGFALS
222	P37802;P37802-2	Transgelin-2	TAGLN2
223	P43251-4;P43251;P43251-3;P	Biotinidase	BTD
224	P43304-2;P43304	Glycerol-3-phosphate dehydrogenas	GPD2
225	P43652;CON__REFSEQ:XP_58	Afamin	AFM
226	P47755	F-actin-capping protein subunit alph	CAPZA2
227	P48735-2;P48735	Isocitrate dehydrogenase [NADP], m	IDH2
228	P50148;O95837	Guanine nucleotide-binding protein	GNAQ
229	P50395-2;P50395	Rab GDP dissociation inhibitor beta	GDI2
230	P50995-2;P50995	Annexin A11	ANXA11
231	P51149	Ras-related protein Rab-7a	RAB7A
232	P51884	Lumican	LUM
233	P52565;P52565-2	Rho GDP-dissociation inhibitor 1	ARHGDIA
	P52566	Rho GDP-dissociation inhibitor 2	ARHGDIB
	P52907	F-actin-capping protein subunit alph	CAPZA1
	P55056	Apolipoprotein C-IV	APOC4
	P55058-3;P55058-4;P55058-2	Phospholipid transfer protein	PLTP
	P55072	Transitional endoplasmic reticulum	VCP
	P55209-3;P55209-2;P55209	Nucleosome assembly protein 1-like	NAP1L1
	P60033	CD81 antigen	CD81
	P60174;P60174-1;P60174-4	Triosephosphate isomerase	TPI1
	P60709;P63261;Q9BYX7	Actin, cytoplasmic 1;Actin, cytoplasn	ACTB;ACTG1
	P61106	Ras-related protein Rab-14	RAB14
	P61158	Actin-related protein 3	ACTR3
	P61224;P61224-3;P61224-4;A	Ras-related protein Rap-1b;Ras-relat	RAP1B

P61626	Lysozyme C	LYZ
P62258-2;P62258	14-3-3 protein epsilon	YWHAE
P62805	Histone H4	HIST1H4A
P62873;P62873-2	Guanine nucleotide-binding protein	GNB1
P62879;P62879-2	Guanine nucleotide-binding protein	GNB2
P62937;P62937-2	Peptidyl-prolyl cis-trans isomerase A	PPIA
P63104;P63104-2	14-3-3 protein zeta/delta	YWHAZ
P67936;P67936-2	Tropomyosin alpha-4 chain	TPM4
P68104-2;Q5VTE0;P68104	Elongation factor 1-alpha 1;Putative	EEF1A1;EEF1A1P5
P68133;P68032;P63267;P627	Actin, alpha skeletal muscle;Actin, al	ACTA1;ACTC1;ACTG2;ACTA2
P68366-2;P68366	Tubulin alpha-4A chain	TUBA4A
P68871	Hemoglobin subunit beta;LVV-hemo	HBB
P69905	Hemoglobin subunit alpha	HBA1
Q00325-2;Q00325	Phosphate carrier protein, mitochon	SLC25A3
Q00610-2;Q00610;P53675-2;	Clathrin heavy chain 1	CLTC
Q01518-2;Q01518	Adenylyl cyclase-associated protein	CAP1
Q02413;Q02413-2	Desmoglein-1	DSG1
Q06033;Q06033-2;CON__Q0	Inter-alpha-trypsin inhibitor heavy c	ITIH3
Q08188	Protein-glutamine gamma-glutamylt	TGM3
Q08380	Galectin-3-binding protein	LGALS3BP
Q08431-3;Q08431-4;Q08431;	Lactadherin;Lactadherin short form;	MFGE8
Q08554-2;Q08554	Desmocollin-1	DSC1
Q12913;Q12913-2	Receptor-type tyrosine-protein phos	PTPRJ
Q13201;Q13201-2	Multimerin-1;Platelet glycoprotein I	MMRN1
Q13418;Q13418-2;Q13418-3	Integrin-linked protein kinase	ILK
Q13576;Q13576-2	Ras GTPase-activating-like protein IC	IQGAP2
Q13790	Apolipoprotein F	APOF
Q14019	Coactosin-like protein	COTL1
Q14165	Malectin	MLEC
Q14344;Q14344-2	Guanine nucleotide-binding protein	GNA13
Q14520-2;Q14520	Hyaluronan-binding protein 2;Hyalur	HABP2
Q14624-3;Q14624-2;Q14624;	Inter-alpha-trypsin inhibitor heavy c	ITIH4
Q14766;Q14766-4;Q14766-3;	Latent-transforming growth factor b	LTBP1
Q15404-2;Q15404	Ras suppressor protein 1	RSU1
Q15431	Synaptonemal complex protein 1	SYCP1
Q15485-2;Q15485	Ficolin-2	FCN2
Q15517	Corneodesmosin	CDSN
Q15582	Transforming growth factor-beta-inc	TGFBI
Q15758;Q15758-3;Q15758-2	Neutral amino acid transporter B(0)	SLC1A5
Q15762	CD226 antigen	CD226
Q15907;Q15907-2;P62491-2;	Ras-related protein Rab-11B;Ras-rel	RAB11B;RAB11A
Q16610;Q16610-2;Q16610-4;	Extracellular matrix protein 1	ECM1
Q4KMQ2-3;Q4KMQ2;Q4KMQ	Anoctamin-6	ANO6
Q6Q788	Apolipoprotein A-V	APOA5
Q7Z406-4;Q7Z406;Q7Z406-6;	Myosin-14	MYH14
Q86UX7-2;Q86UX7;Q9BQL6-3	Fermitin family homolog 3	FERMT3
Q8TDL5;Q8TDL5-2	BPI fold-containing family B member	BPIFB1
Q8WWI5-3;Q8WWI5-2;Q8WV	Choline transporter-like protein 1	SLC44A1
Q8WWZ8	Oncoprotein-induced transcript 3 pr	OIT3
Q92954-5;Q92954-6;Q92954-	Proteoglycan 4;Proteoglycan 4 C-ter	PRG4

Q93084-4;Q93084-2;Q93084-	Sarcoplasmic/endoplasmic reticulum	ATP2A3
Q96DZ9-4;Q96DZ9-5;Q96DZ9-	CKLF-like MARVEL transmembrane d	CMTM5
Q96IY4;Q96IY4-2	Carboxypeptidase B2	CPB2
Q96KN2	Beta-Ala-His dipeptidase	CNDP1
Q96P63;Q96P63-2	Serpin B12	SERPINB12
Q96PD5-2;Q96PD5;CON__EN:	N-acetylmuramoyl-L-alanine amidase	PGLYRP2
Q9H0U4;Q92928	Ras-related protein Rab-1B	RAB1B
Q9H299	SH3 domain-binding glutamic acid-ri	SH3BGRL3
Q9H4M9	EH domain-containing protein 1	EHD1
Q9HBI1-3;Q9HBI1;Q9HBI1-2;C	Beta-parvin	PARVB
Q9UBW5;Q9UBW5-3;Q9UBW	Bridging integrator 2	BIN2
Q9UGM5;Q9UGM5-2	Fetuin-B	FETUB
Q9UHG3;Q9UHG3-2	Prenylcysteine oxidase 1	PCYOX1
Q9UK55	Protein Z-dependent protease inhibi	SERPINA10
Q9ULV4;Q9ULV4-2;Q9ULV4-3	Coronin-1C	CORO1C
Q9Y277;Q9Y277-2	Voltage-dependent anion-selective c	VDAC3
Q9Y490;Q9Y4G6	Talin-1	TLN1
Q9Y613	FH1/FH2 domain-containing protein	FHOD1
Q9Y624;Q9Y624-2	Junctional adhesion molecule A	F11R

100K = 233 identified proteins

	Protein IDs	Protein names	T: Gene names
1	A0A075B6J9	Immunoglobulin lambda variable 2-1	IGLV2-18
2	A0A075B6K4	Immunoglobulin lambda variable 3-1	IGLV3-10
3	A0A087WW87	Immunoglobulin kappa variable 2-40	IGKV2-40
4	A0A0A0MRZ8	Immunoglobulin kappa variable 3D-1	IGKV3D-11
5	A0A0A0MS15	Immunoglobulin heavy variable 3-49	IGHV3-49
6	A0A0B4J1U7	Immunoglobulin heavy variable 6-1	IGHV6-1
7	A0A0B4J1Y9	Immunoglobulin heavy variable 3-72	IGHV3-72
8	A0A0C4DH31	Immunoglobulin heavy variable 1-18	IGHV1-18
9	A0A0C4DH38	Immunoglobulin heavy variable 5-51	IGHV5-51
10	A0A0C4DH43	Immunoglobulin heavy variable 2-70	IGHV2-70D
11	A0A0C4DH67	Immunoglobulin kappa variable 1-8	IGKV1-8
12	A0A0C4DH68	Immunoglobulin kappa variable 2-24	IGKV2-24
13	A0A0J9YX35	Immunoglobulin heavy variable 3-64	IGHV3-64D
14	O00187	Mannan-binding lectin serine protea	MASP2
15	O14791	Apolipoprotein L1	APOL1
16	O43866	CD5 antigen-like	CD5L
17	O75636	Ficolin-3	FCN3
18	O95445	Apolipoprotein M	APOM
19	P00450	Ceruloplasmin	CP
20	P00488	Coagulation factor XIII A chain	F13A1
21	P00734	Prothrombin	F2
22	P00736	Complement C1r subcomponent	C1R
23	P00738	Haptoglobin	HP
24	P00739	Haptoglobin-related protein	HPR
25	P00740	Coagulation factor IX	F9
26	P00747	Plasminogen	PLG
27	P00748	Coagulation factor XII	F12
28	P00751	Complement factor B	CFB

29	P01008	Antithrombin-III	SERPINC1
30	P01009	Alpha-1-antitrypsin	SERPINA1
31	P01011	Alpha-1-antichymotrypsin	SERPINA3
32	P01019	Angiotensinogen	AGT
33	P01023	Alpha-2-macroglobulin	A2M
34	P01024	Complement C3	C3
35	P01031	Complement C5	C5
36	P01034	Cystatin-C	CST3
37	P01040	Cystatin-A	CSTA
38	P01042	Kininogen-1	KNG1
39	P01591	Immunoglobulin J chain	IGJ
40	P01594	Immunoglobulin kappa variable 1-33	IGKV1-33
41	P01599	Immunoglobulin kappa variable 1-17	IGKV1-17
42	P01619	Immunoglobulin kappa variable 3-20	IGKV3-20
43	P01701	Immunoglobulin lambda variable 1-5	IGLV1-51
44	P01703	Immunoglobulin lambda variable 1-4	IGLV1-40
45	P01743	Immunoglobulin heavy variable 1-46	IGHV1-46
46	P01782	Immunoglobulin heavy variable 3-9	IGHV3-9
47	P01833	Polymeric immunoglobulin receptor	PIGR
48	P01834	Immunoglobulin kappa constant	IGKC
49	P01859	Immunoglobulin heavy constant gam	IGHG2
50	P01860	Immunoglobulin heavy constant gam	IGHG3
51	P01861	Immunoglobulin heavy constant gam	IGHG4
52	P01871	Immunoglobulin heavy constant mu	IGHM
53	P01876	Immunoglobulin heavy constant alpl	IGHA1
54	P02647	Apolipoprotein A-I	APOA1
55	P02649	Apolipoprotein E	APOE
56	P02652	Apolipoprotein A-II	APOA2
57	P02654	Apolipoprotein C-I	APOC1
58	P02655	Apolipoprotein C-II	APOC2
59	P02656	Apolipoprotein C-III	APOC3
60	P02671	Fibrinogen alpha chain	FGA
61	P02675	Fibrinogen beta chain	FGB
62	P02679	Fibrinogen gamma chain	FGG
63	P02730	Band 3 anion transport protein	SLC4A1
64	P02741	C-reactive protein	CRP
65	P02743	Serum amyloid P-component	APCS
66	P02745	Complement C1q subcomponent sul	C1QA
67	P02746	Complement C1q subcomponent sul	C1QB
68	P02747	Complement C1q subcomponent sul	C1QC
69	P02748	Complement component C9	C9
70	P02749	Beta-2-glycoprotein 1	APOH
71	P02750	Leucine-rich alpha-2-glycoprotein	LRG1
72	P02751	Fibronectin	FN1
73	P02753	Retinol-binding protein 4	RBP4
74	P02760	Protein AMBP	AMBP
75	P02763	Alpha-1-acid glycoprotein 1	ORM1
76	P02765	Alpha-2-HS-glycoprotein	AHSG
77	P02766	Transthyretin	TTR
78	P02774	Vitamin D-binding protein	GC

79	P02776	Platelet factor 4	PF4
80	P02786	Transferrin receptor protein 1	TFRC
81	P02787	Serotransferrin	TF
82	P02788	Lactotransferrin	LTF
83	P02790	Hemopexin	HPX
84	P02792	Ferritin light chain	FTL
85	P03951	Coagulation factor XI	F11
86	P03952	Plasma kallikrein	KLKB1
87	P04003	C4b-binding protein alpha chain	C4BPA
88	P04004	Vitronectin	VTN
89	P04075	Fructose-bisphosphate aldolase A	ALDOA
90	P04114	Apolipoprotein B-100	APOB
91	P04180	Phosphatidylcholine-sterol acyltrans	LCAT
92	P04196	Histidine-rich glycoprotein	HRG
93	P04217	Alpha-1B-glycoprotein	A1BG
94	P04275	von Willebrand factor	VWF
95	P04406	Glyceraldehyde-3-phosphate dehydr	GAPDH
96	P04430	Immunoglobulin kappa variable 1-16	IGKV1-16
97	P04792	Heat shock protein beta-1	HSPB1
98	P05090	Apolipoprotein D	APOD
99	P05106	Integrin beta-3	ITGB3
100	P05109	Protein S100-A8	S100A8
101	P05154	Plasma serine protease inhibitor	SERPINA5
102	P05155	Plasma protease C1 inhibitor	SERPING1
103	P05156	Complement factor I chain	CFI
104	P05160	Coagulation factor XIII B chain	F13B
105	P05452	Tetranectin	CLEC3B
106	P05543	Thyroxine-binding globulin	SERPINA7
107	P05546	Heparin cofactor 2	SERPIND1
108	P06312	Immunoglobulin kappa variable 4-1	IGKV4-1
109	P06396	Gelsolin	GSN
110	P06681	Complement C2 fragment	C2
111	P06702	Protein S100-A9	S100A9
112	P06727	Apolipoprotein A-IV	APOA4
113	P06733	Alpha-enolase	ENO1
114	P07225	Vitamin K-dependent protein S	PROS1
115	P07339	Cathepsin D	CTSD
116	P07355	Annexin A2	ANXA2
117	P07357	Complement component C8 alpha cl	C8A
118	P07358	Complement component C8 beta ch	C8B
119	P07360	Complement component C8 gamma	C8G
120	P08185	Corticosteroid-binding globulin	SERPINA6
121	P08514	Integrin alpha-Iib	ITGA2B
122	P08519	Apolipoprotein(a)	LPA
123	P08571	Monocyte differentiation antigen CD	CD14
124	P08603	Complement factor H	CFH
125	P08697	Alpha-2-antiplasmin	SERPINF2
126	P09871	Complement C1s subcomponent	C1S
127	P0C0L4	Complement C4-A	C4A
128	P0C0L5	Complement C4-B	C4B

129	Q71UI9	Histone H2A.V	H2AZ2
130	P0DJI8	Serum amyloid A-1 protein	SAA1
131	P0DJI9	Serum amyloid A-2 protein	SAA2
132	P0DOX2	Immunoglobulin alpha-2 heavy chair	IGHA2
133	P0DOX3	Immunoglobulin delta heavy chain	IGHD
134	P01857	Ig gamma-1 chain C region	IGHG1
135	P0DOX6	Immunoglobulin mu heavy chain	IGHM(2)
136	P0DOX7	Immunoglobulin kappa light chain	IGKC(2)
137	B9A064	Immunoglobulin lambda-like polype	IGLL5
138	P0CF74	Ig lambda-6 chain C region	IGLC6
139	P10599	Thioredoxin	TXN
140	P10643	Complement component C7	C7
141	P10909	Clusterin	CLU
142	P11021	Endoplasmic reticulum chaperone Bi	HSPA5
143	P11142	Heat shock cognate 71 kDa protein	HSPA8
144	P11166	Solute carrier family 2	SLC2A1
145	P11532	Dystrophin	DMD
146	P11597	Cholesteryl ester transfer protein	CETP
147	P12259	Coagulation factor V	F5
148	P12273	Prolactin-inducible protein	PIP
149	P13671	Complement component C6	C6
150	P14923	Junction plakoglobin	JUP
151	P15144	Aminopeptidase N	ANPEP
152	P15814	Immunoglobulin lambda-like polype	IGLL1
153	P15924	Desmoplakin	DSP
154	P16671	Platelet glycoprotein 4	CD36
155	P18428	Lipopolysaccharide-binding protein	LBP
156	P19440	Glutathione hydrolase 1 proenzyme	GGT1
157	P19652	Alpha-1-acid glycoprotein 2	ORM2
158	P19823	Inter-alpha-trypsin inhibitor heavy cl	ITIH2
159	P19827	Inter-alpha-trypsin inhibitor heavy cl	ITIH1
160	P20073	Annexin A7	ANXA7
161	Q9NRW1	Ras-related protein Rab-6B	RAB6B
162	P20851	C4b-binding protein beta chain	C4BPB
163	P21333	Filamin-A	FLNA
164	P22352	Glutathione peroxidase 3	GPX3
165	P22792	Carboxypeptidase N subunit 2	CPN2
166	P23083	Immunoglobulin heavy variable 1-2	IGHV1-2
167	P23142	Fibulin-1	FBLN1
168	P25311	Zinc-alpha-2-glycoprotein	AZGP1
169	P27105	Erythrocyte band 7 integral membra	STOM
170	P27169	Serum paraoxonase/arylesterase 1	PON1
171	P27918	Properdin	CFP
172	P29508	Serpin B3	SERPINB3
173	P29622	Kallistatin	SERPINA4
174	P29972	Aquaporin-1	AQP1
175	P31151	Protein S100-A7	S100A7
176	P31944	Caspase-14	CASP14
177	P35542	Serum amyloid A-4 protein	SAA4
178	P35579	Myosin-9	MYH9

179	P35858	Insulin-like growth factor-binding pro acid labile subunit	IGFALS
180	P36955	Pigment epithelium-derived factor	SERPINF1
181	P36980	Complement factor H-related protei	CFHR2
182	P43652	Afamin	AFM
183	P48740	Mannan-binding lectin serine protea	MASP1
184	P49908	Selenoprotein P	SEPP1
185	P51884	Lumican	LUM
186	P55056	Apolipoprotein C-IV	APOC4
187	P55058	Phospholipid transfer protein	PLTP
188	P60660	Myosin light polypeptide 6	MYL6
189	P60709	Actin, cytoplasmic 1	ACTB
190	P61224	Ras-related protein Rap-1b	RAP1B
191	P61626	Lysozyme C	LYZ
192	P62805	Histone H4	HIST1H4A
193	P62937	Peptidyl-prolyl cis-trans isomerase A	PPIA
194	P62979	Ubiquitin-40S ribosomal protein S27	RPS27A
195	P63104	14-3-3 protein zeta/delta	YWHAZ
196	P68133	Actin, alpha skeletal muscle	ACTA1
197	P68871	Hemoglobin subunit beta	HBB
198	P69905	Hemoglobin subunit alpha	HBA1
199	P78509	Reelin	RELN
200	P80108	Phosphatidylinositol-glycan-specific	GPLD1
201	A0A075B6K5	Immunoglobulin lambda variable 3-9	IGLV3-9
202	P81605	Dermcidin	DCD
203	Q01469	Fatty acid-binding protein 5	FABP5
204	Q02413	Desmoglein-1	DSG1
205	Q06033	Inter-alpha-trypsin inhibitor heavy cl	ITIH3
206	Q06830	Peroxiredoxin-1	PRDX1
207	Q08188	Protein-glutamine gamma-glutamylt	TGM3
208	Q08380	Galectin-3-binding protein	LGALS3BP
209	Q08554	Desmocollin-1	DSC1
210	Q14520	Hyaluronan-binding protein 2	HABP2
211	Q14624	Inter-alpha-trypsin inhibitor heavy cl	ITIH4
212	Q15485	Ficolin-2	FCN2
213	Q15517	Corneodesmosin	CDSN
214	Q15582	Transforming growth factor-beta-inc ig-h3	TGFBI
215	Q16610	Extracellular matrix protein 1	ECM1
216	Q4LDE5	Sushi, von Willebrand factor type A, pentraxin domain-containing protein 1	SVEP1
217	Q6Q788	Apolipoprotein A-V	APOA5
218	Q86UX7	Fermitin family homolog 3	FERMT3
219	Q8IZ83	Aldehyde dehydrogenase family 16 r	ALDH16A1
220	Q8TDL5	BPI fold-containing family B member	BPIFB1
221	Q8WWZ8	Oncoprotein-induced transcript 3 pro	OIT3
222	Q92496	Complement factor H-related protei	CFHR4
223	Q92954	Proteoglycan 4	PRG4
224	Q96IY4	Carboxypeptidase B2	CPB2
225	Q96KN2	Beta-Ala-His dipeptidase	CNDP1

226	Q96P63	Serpin B12	SERPINB12
227	Q96PD5	N-acetylmuramoyl-L-alanine amidas	PGLYRP2
228	Q9BWP8	Collectin-11	COLEC11
229	Q9BXR6	Complement factor H-related protei	CFHR5
230	Q9UGM5	Fetuin-B	FETUB
231	Q9UHG3	Prenylcysteine oxidase 1	PCYOX1
232	Q9Y490	Talin-1	TLN1
233	Q9Y6R7	IgGFc-binding protein	FCGBP

	**Vesiclepedia Top 100**	**ExoCarta Top 100**
	(accessed 03.12.20)	(accessed 03.12.20)
1	PDCD6IP	CD9
2	GAPDH	HSPA8
3	HSPA8	PDCD6IP
4	ACTB	GAPDH
5	ANXA2	ACTB
6	CD9	ANXA2
7	PKM	CD63
8	HSP90AA1	SDCBP
9	ENO1	ENO1
10	ANXA5	HSP90AA1
11	HSP90AB1	TSG101
12	CD63	PKM
13	YWHAZ	LDHA
14	YWHAE	EEF1A1
15	EEF1A1	YWHAZ
16	PGK1	PGK1
17	CLTC	EEF2
18	PPIA	ALDOA
19	SDCBP	HSP90AB1
20	ALDOA	ANXA5
21	EEF2	FASN
22	ALB	YWHAE
23	TPI1	CLTC
24	VCP	CD81
25	CFL1	ALB
26	MSN	VCP
27	ATP1A1	TPI1
28	PRDX1	PPIA
29	MYH9	MSN
30	EZR	CFL1
31	CD81	PRDX1
32	ANXA6	PFN1
33	FLOT1	RAP1B
34	YWHAB	ITGB1
35	LDHB	HSPA5
36	SLC3A2	SLC3A2
37	GNB1	HIST1H4A
38	PFN1	GNB2
39	TSG101	ATP1A1

40	YWHAQ	YWHAQ
41	GNAI2	FLOT1
42	CLIC1	FLNA
43	ANXA1	CLIC1
44	ITGB1	CDC42
45	LDHA	CCT2
46	FASN	A2M
47	CDC42	YWHAG
48	RAP1B	TUBA1B
49	CCT2	RAC1
50	YWHAG	LGALS3BP
51	GNB2	HSPA1A
52	ACTN4	GNAI2
53	RAB5C	ANXA1
54	C3	RHOA
55	RAB10	MFGE8
56	HIST1H4A	PRDX2
57	KRT1	GDI2
58	FN1	EHD4
59	AHCY	ACTN4
60	A2M	YWHAB
61	BSG	RAB7A
62	ACTN1	LDHB
63	ANXA7	GNAS
64	ACLY	TFRC
65	HIST1H4B	RAB5C
66	GDI2	ARF1
67	FLNA	ANXA6
68	UBA1	ANXA11
69	GNAS	ACTG1
70	GSN	KPNB1
71	CCT4	EZR
72	RAN	ANXA4
73	PRDX2	ACLY
74	RHOA	TUBA1C
75	CCT3	RAB14
76	RAC1	HIST2H4A
77	LGALS3BP	GNB1
78	TCP1	UBA1
79	KRT10	THBS1
80	CAP1	RAN
81	RAB7A	RAB5A
82	TUBB4B	PTGFRN
83	HSPA5	CCT5
84	IQGAP1	CCT3
85	GPI	BSG
86	RALA	AHCY
87	KPNB1	RAB5B
88	HIST1H4I	RAB1A
89	TFRC	LAMP2

90	EIF4A1	ITGA6	
91	HIST4H4	HIST1H4B	
92	CCT8	GSN	
93	TLN1	FN1	
94	HIST1H4K	YWHAH	
95	HIST1H4H	TUBA1A	
96	CCT6A	TKT	
97	ANXA11	TCP1	
98	HIST1H4J	STOM	
99	HIST1H4F	SLC16A1	
100	HIST1H4D	RAB8A	

	**20K Venn**		
	**Common in Study, Exocarta & Vesiclepedia (40)**	**Exocarta vs study (6)**	**Study vs vesiclepedia (5)**
1	CLIC1	THBS1	C3
2	ACTN4	LAMP2	ANXA7
3	LDHA	ITGA6	MYH9
4	PGK1	STOM	CAP1
5	A2M	RAB14	TLN1
6	FN1	MFGE8	
7	TFRC		
8	ALDOA		
9	ANXA1		
10	GAPDH		
11	GNAI2		
12	ATP1A1		
13	ITGB1		
14	GSN		
15	ENO1		
16	PFN1		
17	HSP90AA1		
18	ANXA6		
19	HSPA5		
20	HSPA8		
21	PKM		
22	FLNA		
23	CFL1		
24	MSN		
25	PRDX2		
26	GDI2		
27	ANXA11		
28	RAB7A		
29	VCP		
30	CD81		
31	TPI1		
32	RAP1B		
33	YWHAE		
34	HIST1H4A		
35	GNB1		

36	GNB2
37	PPIA
38	YWHAZ
39	CLTC
40	LGALS3BP

	**100K Venn**		
	**Common in Study, Exocarta & Vesiclepedia (18)**	**Exocarta vs study (1)**	**Study vs. Vesiclepedia (4)**
1	A2M	STOM	C3
2	FN1		ACTN1
3	TFRC		MYH9
4	ALDOA		TLN1
5	GAPDH		
6	GSN		
7	ENO1		
8	ANXA2		
9	HSPA5		
10	HSPA8		
11	FLNA		
12	ACTB		
13	RAP1B		
14	HIST1H4A		
15	PPIA		
16	YWHAZ		
17	PRDX1		
18	LGALS3BP		
