# Supplementary material for: Circulating microvesicles and exosomes in small cell lung cancer by quantitative proteomics
Source: Clin Proteomics. 2022 Jan 7;19:2. doi: 10.1186/s12014-021-09339-5 (PMC8903681; doi:10.1186/s12014-021-09339-5)
Supplement: Supplementary file 3 — Additional file 3: Table S2. Protein clusters and gene names. [file 12014_2021_9339_MOESM3_ESM.pdf]

Table S2. An overview of the proteins identified in each cluster. For the **20K sample,** proteins were clustered in 10 distinct clusters based on protein expression patterns in the two groups; control and SCLC patients. For the **100K sample,** proteins were clustered in 12 distinct clusters based on protein expression patterns in the two groups; control and SCLC patients. The proteins are listed in terms of their gene names.

20K

**Cluster 1 (n = 42)**			
SERPINA6	PRG4	CDSN	HRG
ITGAL	SERPINC1	AZGP1	C8B
C4BPB	ITIH3	SERPINA7	SERPINB12
LBP	IGHV1-46	C8A	CFH
SERPING1	IGKV1-33	F10	SERPINF2
IGHV3-49	SYCP1	F7	FCN2
C1QA	CPN2	TGM3	JUP
APOC1	DSC1	IGHM	SERPIND1
AGT	APOL1	ANXA2	IGHV5-51
ECM1	IGKC	ITIH4	
IGHV2-26	IGK	BTD	
**Cluster 2 (n = 26)**			
SPTA1	IGHG3	HBA1	FN1
CA1	GYPA	F2	LCAT
HBD	SLC4A1	APOH	ANXA6
CD81	PLTP	LCP1	CAT
F11	HLA-DRA	GSN	BPIFB1
PLG	SPTB	ICAM1	
ANK1	HBB	APOC3	
**Cluster 3 (n = 40)**			
CSTA	C1QB	C7	APOA4
APOE	C1S	CNDP1	KLKB1
GPX3	TGFBI	AMBP	C4B
F12	PRDX2	APOF	IGHV3-23
ITIH2	PGLYRP2	HPR	C1R
SERPINA5	QSOX1	DSP	ITIH1
PIP	TTR	IGFALS	FETUB
VTN	RBP4	PROS1	ATRN
KNG1	HSPB1	CD5L	SERPINA4
F13B	GC	LUM	CLEC3B
**Cluster 4 (n = 22)**			
IGHA1	IGHG2	CLU	C4BPA
APOA1	TF	DSG1	IGJ
C8G	FGG	IGLC6	HABP2
CETP	IGKV3-20	SERPINA10	SHBG
AHSG	FGB	C6	
PON1	CD14	FGA	
**Cluster 5 (n = 27)**			
CFB	APOM	A1BG	SAA1
IGHV3-9	S100A7	HPX	LGALS3BP

PIGR	APOB	CRP	CFI
C5	ITGB2	BCHE	CASP14
S100A9	PCYOX1	C4A	ORM1
SERPINA1	ANPEP	HP	A2M
IGHG4	ORM2	C2	
**Cluster 6 (n = 14)**			
CP	C3	CTSD	IGM
SAA4	SERPINA3	ANXA1	C9
IGHV1-24	PZP	ARG1	
LTF	LYZ	IGHG1	
**Cluster 7 (n = 13)**			
CAPZA2	SLC2A1	LTBP1	OIT3
TPI1	ACTA1	COTL1	
ALDOC	GDI2	GNB1	
ACTN4	MSN	EEF1A1	
**Cluster 8 (n = 32)**			
RAB27B	GNAI2	GP1BA	ANXA11
MMRN1	RAB7A	ITGA2	ILK
CPN1	ITGA2B	LYN	APOA5
PTPRJ	HLA-A	ICAM2	LPA
TFRC	PPIA	FERMT3	LAMP2
HSPD1	VCP	RAB14	LRG1
AFM	ITGA6	FCN3	MPO
PLEK	RSU1	CPB2	LDHA
**Cluster 9 (n = 55)**			
PNP	CD226	APOC4	HSP90AA1
FBLN1	ACTB	CAPZA1	SH3BGRL3
MYH14	PKM	TAGLN2	YWHAZ
MYH9	ARHGDIA	FHOD1	ATP5A1
CALM3	ACTN1	RAB1B	TUBB
CAP1	ARHGDIB	GAPDH	IQGAP2
CLTC	ENO1	NAP1L1	GPD2
FTL	CORO1C	PRDX6	GNB2
YWHAE	GNA13	MYL12A	TUBA4A
CLIC1	BIN2	TPM4	G6B
CFL1	F13A1	RHOC	WDR1
SLC44A1	ALDOA	PFN1	CYB5R3
PGK1	SLC25A5	HSPA5	MLEC
PTPRC	F11R	ANO6	
**Cluster 10 (n = 43)**			
CPNE	ATP5B	CANX	RAP1B
F5	PF4	SLC25A3	VCL
MFGE8	HSPA8	CMTM5	TLN1
EHD1	SLC1A5	SDPR	PARVB
HLA-C	FLNA	ATP1A1	VWF
GP1BB	RAB11B	SLC2A3	THBS1
HSPA1B	CS	GNAQ	GP9

VDAC3	PECAM1	GSTP1	CD36
ACTR3	CAPN1	ITGB3	PDIA3
HIST1H4A	ATP2A3	HLA-B	ITGB1
IDH2	STOM	SERPINB1	

100K

**Cluster 1 (n = 12)**			
HSPA5	OIT3	A1BG	PPIA
RBP4	F13A1	ACTA1	TTR
HBA1	F13B	ALDOA	A2M
**Cluster 2 (n = 13)**			
FABP5	FN1	FETUB	SERPINF1
JUP	APOH	ANXA2	
LUM	ACTB	SLC4A1	
HBB	S100A7	SERPINA5	
**Cluster 3 (n = 37)**			
ITIH2	C4A	AHSG	HSPB1
APOM	IGHG1	F11	FGG
IGFALS	IGHV3-9	IGHG2	FCGBP
FGB	IGLV3-9	MASP1	C8B
IGKV4-1	IGHG3	SERPINA4	FCN2
HRG	C1S	GSN	C1R
FCN3	TF	ITIH1	IGHV3-49
SERPING1	HPR	COLEC11	
FGA	CETP	CLEC3B	
PRDX1	IGHV5-51	SERPINA6	
**Cluster 4 (n = 30)**			
SEPP1	C1QB	RELN	IGKV2-24
ENO1	SERPIND1	PGLYRP2	KLKB1
TGFBI	IGHM	CD5L	IGKV3D-11
APOA4	GC	C1QA	C4BPA
IGM	IGKV3-20	IGHA1	IGHV3-64D
IGKV1-16	IGJ	IGKV1-8	IGKV2-40
C1QC	PON1	PLTP	
IGLV1-51	IGLL5	IGHV6-1	
**Cluster 5 (n = 15)**			
IGA2	SVEP1	IGLC6	KNG1
CNDP1	APOA1	PROS1	CPN2
IGLV1-40	ITIH4	C4B	LGALS3BP
SERPINC1	IGKV1-17	APOL1	
**Cluster 6 (n = 6)**			
IGK	IGKC	IGLL1	
CLU	C4BPB	F12	
**Cluster 7 (n = 20)**			
MYH9	LYZ	TLN1	DSP

TFRC	STOM	SERPINB3	CST3
HSPA8	SAA4	FBLN1	APOC1
AQP1	F5	APOC4	FTL
IGHV1-2	RAB6B	SERPINA7	APOE
**Cluster 8 (n = 11)**			
AFM	LCAT	PLG	YWHAZ
FLNA	SLC2A1	VWF	ECM1
CTSD	SERPINF2	GPX3	
**Cluster 9 (n = 23)**			
TXN	DSG1	SERPINB12	C5
CFHR4	RAP1B	PCYOX1	ITGB3
MYL6	CFH	APOA2	APOD
CASP14	RPS27A	CSTA	APOB
CD36	CDSN	PF4	HPX
TGM3	ITGA2B	HABP2	
**Cluster 10 (n = 45)**			
PRG4	APOC3	C6	CRP
H2AFV	APOC2	LPA	CPB2
GGT1	DCD	FERMT3	CP
C8A	ANXA7	HP	IGHV1-18
CFHR2	GPLD1	IGD	PIP
SAA2	ALDH16A1	SAA1	HIST1H4A
AMBP	APOA5	C9	C2
C7	DSC1	GAPDH	ORM1
PIGR	ITIH3	IGKV1-33	APCS
SERPINA3	LRG1	C3	
CFP	BPIFB1	IGHV2-70D	
CFB	CFI	ANPEP	
**Cluster 11 (n = 13)**			
AZGP1	IGHV1-46	F2	IGLV3-10
CFHR5	LBP	VTN	
LTF	MASP2	AGT	
DMD	SERPINA1	S100A8	
**Cluster 12 (n = 8)**			
F9	CD14	IGHG4	C8G
ORM2	S100A9	IGHV3-72	IGLV2-18
