# Supplementary material for: Circulating microvesicles and exosomes in small cell lung cancer by quantitative proteomics
Source: Clin Proteomics. 2022 Jan 7;19:2. doi: 10.1186/s12014-021-09339-5 (PMC8903681; doi:10.1186/s12014-021-09339-5)
Supplement: Supplementary file 4 — Additional file 4: Table S3. Functional Enrichment analysis. [file 12014_2021_9339_MOESM4_ESM.pdf]

Table S3: Functional enrichment analysis performed on the significantly differentially expressed proteins. *The significantly differentially expressed proteins were investigated for enrichment of Gene Ontology Biological Processes (GOBP) terms in order to identify biological processes related to the up- and downregulated proteins in SCLC patients for 20K and 100K. The tables depict the GOBP terms related to the up- or downregulated proteins and the corresponding Enrichment Score (ES), p-value, and q-value. Abbreviations – SCLC: Small cell lung cancer, GOBP: Gene Ontology Biological Processes, ES: Enrichment Score.*

**↑20K SCLC**			
**GOBP term**	***ES***	**p-value**	**q-value**
Cell-matrix adhesion	3.18	0.0003	0.0280
Integrin-mediated signaling pathway	3.18	0.0004	0.0280
Cell adhesion	3.18	0.0005	0.0280
Extracellular matrix organization	3.18	0.0030	0.1200
**↑100K SCLC**			
**GOBP term**	***ES***	**p-value**	**q-value**
Regulation of complement activation	4.95	<0.0001	<0.0001
Complement activation, alternative pathway	4.95	<0.0001	<0.0001
Complement activation	4.95	0.0004	0.00980
Cytolysis	4.95	0.0006	0.0120
Complement activation, classical pathway	4.95	0.0006	0.0120
Lipid transport	4.56	<0.0001	<0.0001
Lipoprotein metabolic process	4.56	<0.0001	<0.0001
Triglyceride-rich lipoprotein particle remodelling	4.56	<0.0001	0.0003
Cholesterol efflux	4.56	<0.0001	0.0003
Cholesterol homeostasis	4.56	0.0002	0.0052
Phospholipid efflux	4.56	0.0002	0.0070
Reverse cholesterol transport	4.56	0.0004	0.0098
Retinoid metabolic process	4.56	0.0046	0.0790
**↓ 20K SCLC (no enrichment)**			
**GOBP term**	***ES***	**p-value**	**q-value**
Platelet degranulation	–	<0.0001	<0.0001
Negative regulation of endopeptidase activity	–	<0.0001	<0.0001
Blood coagulation	–	<0.0001	0.0006
Hydrogen peroxide catabolic process	–	<0.0001	0.0006
Extracellular matrix disassembly	–	<0.0001	0.0010
Positive regulation of fibrinolysis	–	<0.0001	0.0011
Blood coagulation	–	<0.0001	0.0015
Bicarbonate transport	–	0.0001	0.0035
Plasminogen activatin	–	0.0002	0.0046
Negative regulation of blood coagulation	–	0.0002	0.0075
Actin filament capping	–	0.0003	0.0081
Oxygen transport	–	0.0004	0.0099
Cellular protein metabolic process	–	0.0019	0.0390
Extracellular matrix organization	–	0.0078	>0.05
Lipid transport	–	0.0110	>0.05
ER to Golgi vesicle-mediated transport	–	0.0440	>0.05
Receptor-mediated endocytosis	–	>0.05	>0.05
Visual perception	–	>0.05	>0.05
Positive regulation of gene expression	–	>0.05	>0.05
Cell adhesion	–	>0.05	>0.05

Proteolysis	–	>0.05	>0.05
**↓ 100K SCLC**			
**GOBP term**	***ES***	**p-value**	**q-value**
Complement activation	7.45	<0.0001	<0.0001
Complement activation, classical pathway	7.45	<0.0001	<0.0001
Proteolysis	7.45	<0.0001	<0.0001
Receptor-mediated endocytosis	7.45	<0.0001	<0.0001
Fc-gamma receptor signaling pathway involved in phagocytosis	7.45	0.0001	0.0020
Immune response	7.45	0.0090	0.1000
Regulation of immune response	7.45	0.0480	0.3600
Fc-epsilon receptor signaling pathway	7.45	0.0480	0.3600
Phagocytosis, engulfment	4.46	<0.0001	<0.0001
Innate immune response	4.46	<0.0001	<0.0001
Positive regulation of B cell activation	4.46	<0.0001	0.0005
Phagocytosis, recognition	4.46	<0.0001	0.0005
B cell receptor signaling pathway	4.46	0.0002	0.0027
Defense response to bacterium	4.46	0.0330	0.2800
