# Supplementary material for: Circulating microvesicles and exosomes in small cell lung cancer by quantitative proteomics
Source: Clin Proteomics. 2022 Jan 7;19:2. doi: 10.1186/s12014-021-09339-5 (PMC8903681; doi:10.1186/s12014-021-09339-5)
Supplement: Supplementary file 5 — Additional file 5: Table S4. Significantly regulated proteins in 20 K and 100 K samples. [file 12014_2021_9339_MOESM5_ESM.pdf]

Table S4. Significantly differentially expressed proteins for 20K and 100K comparing SCLC patients to the control group.

SCLC │ Control (20K pellet)				
**Uniprot ID**	**Gene name**	**Protein name**	**Log_2 FC**	***p*-value**
P02741	CRP	C-reactive protein	3.5	0.0001
P15144	ANPEP	Aminopeptidase N	3.2	0.0004
P0DJI8	SAA1	Serum amyloid A-1 protein	2.4	< 0.0001
P02786	TFRC	Transferrin receptor protein 1	2.2	0.0003
Q08380	LGALS3BP	Galectin-3-binding protein	2.2	0.0008
P05164	MPO	Myeloperoxidase	1.2	0.0424
Q9UHG3	PCYOX1	Prenylcysteine oxidase 1	1.1	0.0447
Q13418	ILK	Integrin-linked protein kinase	1.0	0.0140
P02763	ORM1	Alpha-1-acid glycoprotein 1	1.0	0.0011
P23229	ITGA6	Integrin alpha-6	1.0	0.0193
P04114	APOB	Apolipoprotein B	1.0	0.0019
P00738	HP	Haptoglobin	0.9	0.0004
P50995	ANXA11	Annexin A11	0.9	0.0345
Q13201	MMRN1	Multimerin-1	0.9	0.0136
P02750	LRG1	Leucine-rich alpha-2-glycoprotein	0.9	0.0146
P17301	ITGA2	Integrin alpha-2	0.8	0.0388
P08514	ITGA2B	Integrin alpha-IIb	0.7	0.0163
P51149	RAB7A	Ras-related protein Rab-7a	0.6	0.0432
P00338	LDHA	L-lactate dehydrogenase A chain	0.6	0.0304
P13598	ICAM2	Intercellular adhesion molecule 2	0.6	0.0031
P01011	SERPINA3	Alpha-1-antichymotrypsin	0.5	0.0100
P01009	SERPINA1	Alpha-1-antitrypsin	0.5	0.0147
P06681	C2	Complement C2	0.5	0.0240
P01031	C5	Complement C5	0.5	0.0086
P13473	LAMP2	Lysosome-associated membrane glycoprotein 2	0.5	0.0113
P00450	CP	Ceruloplasmin	0.4	0.0476
P19827	ITIH1	Inter-alpha-trypsin inhibitor heavy chain H1	-0.4	0.0218
P00747	PLG	Plasminogen	-0.4	0.0326
P51884	LUM	Lumican	-0.4	0.0398
Q14019	COTL1	Coactosin-like protein	-0.4	0.0195
P03952	KLKB1	Plasma kallikrein	-0.5	0.0053
P05154	SERPINA5	Plasma serine protease inhibitor	-0.5	0.0201
P04004	VTN	Vitronectin	-0.5	0.0232
P02765	AHSG	Alpha-2-HS-glycoprotein	-0.5	0.0367
P02753	RBP4	Retinol-binding protein 4	-0.6	0.0404

P02749	APOH	Beta-2-glycoprotein 1	-0.6	0.0088
P01042	KNG1	Kininogen-1	-0.6	0.0100
P02751	FN1	Fibronectin	-0.6	0.0034
P55058	PLTP	Phospholipid transfer protein	-0.7	0.0011
Q9UGM5	FETUB	Fetuin-B	-0.7	0.0000
P11166	SLC2A1	Solute carrier family 2	-0.7	0.0540
P13796	LCP1	Plastin-2	-0.8	0.0033
P03951	F11	Coagulation factor XI	-0.8	0.0116
P68133	ACTA1	Actin, alpha skeletal muscle	-0.9	0.0051
P05160	F13B	Coagulation factor XIII B chain	-0.9	< 0.0001
P05452	CLEC3B	Tetranectin	-0.9	< 0.0001
P29622	SERPINA4	Kallistatin	-0.9	0.0014
Q96PD5	PGLYRP2	N-acetylmuramoyl-L-alanine amidase	-1.0	< 0.0001
P06396	GSN	Gelsolin	-1.0	< 0.0001
O00391	QSOX1	Sulfhydryl oxidase 1	-1.1	0.0052
P02724	GYPA	Glycophorin-A	-1.1	0.0046
P06727	APOA4	Apolipoprotein A-IV	-1.1	0.0001
P00915	CA1	Carbonic anhydrase 1	-1.2	0.0028
P32119	PRDX2	Peroxiredoxin-2	-1.2	0.0351
P69905	HBA1	Hemoglobin subunit alpha	-1.2	0.0002
Q15582	TGFBI	Transforming growth factor-beta-induced protein ig-h3	-1.2	< 0.0001
P02730	SLC4A1	Band 3 anion transport protein	-1.6	0.0001
P68871	HBB	Hemoglobin subunit beta	-1.6	< 0.0001
P02042	HBD	Hemoglobin subunit delta	-1.7	< 0.0001
P16157	ANK1	Ankyrin-1	-2,6	0.0233
P11277	SPTB	Spectrin beta chain erythrocytic	-2.7	0.0502
P02549	SPTA1	Spectrin alpha chain erythrocytic 1	-3.2	0.0106
**SCLC │ Control (100K)**				
**Uniprot ID**	**Gene name**	**Protein name**	**Log$_2$ FC**	***p*-value**
P0DJI9	SAA2	Serum amyloid A-2 protein	3.3	0.0016
P0DJI8	SAA1	Serum amyloid A-1 protein	2.9	< 0.0001
P02655	APOC2	Apolipoprotein C-II	2.8	0.0062
P15144	ANPEP	Aminopeptidase N	2.4	0.0006
P08519	LPA	Apolipoprotein(a)	1.4	0.0346
Q92496	CFHR4	Complement factor H-related protein 4	1.2	0.0005
P02741	CRP	C-reactive protein	1.2	0.0467
P02750	LRG1	Leucine-rich alpha-2-glycoprotein	1.2	0.00006
P00738	HP	Haptoglobin	1.2	< 0.0001

P04114	APOB	Apolipoprotein B	1.1	0.00002
Q06033	ITIH3	Inter-alpha-trypsin inhibitor heavy chain H3	0.9	0.0012
P01011	SERPINA3	Alpha-1-antichymotrypsin	0.8	< 0.0001
P02748	C9	Complement component C9	0.7	< 0.0001
Q6Q788	APOA5	Apolipoprotein A-V	0.7	0.0165
P60660	MYL6	Myosin light polypeptide 6	0.7	0.0220
P02652	APOA2	Apolipoprotein A-II	0.7	0.0521
P61224	RAP1B	Ras-related protein Rap-1b	0.6	0.0234
P00751	CFB	Complement factor B	0.6	0.0003
P62979	RPS27A	Ubiquitin-40S ribosomal protein S27a	0.5	0.0427
Q96P63	SERPINB12	Serpin B12	0.5	0.0397
P01040	CSTA	Cystatin-A	0.5	0.0481
Q15517	CDSN	Corneodesmosin	0.5	0.0244
P13671	C6	Complement component C6	0.5	0.0265
Q96IY4	CPB2	Carboxypeptidase B2	0.4	0.0428
P05090	APOD	Apolipoprotein D	0.4	0.0410
P02763	ORM1	Alpha-1-acid glycoprotein 1	0.4	0.0474
P01031	C5	Complement C5	0.3	0.0052
P07357	C8A	Complement component C8 alpha chain	0.3	0.0505
P04004	VTN	Vitronectin	0.3	0.0346
P19827	ITIH1	Inter-alpha-trypsin inhibitor heavy chain H1	-0.3	0.0504
B9A064	IGLL5	Immunoglobulin lambda-like polypeptide 5	-0.4	0.0204
P19823	ITIH2	Inter-alpha-trypsin inhibitor heavy chain H2	-0.5	0.0052
P01619	IGKV3-20	Immunoglobulin kappa variable 3-20	-0.5	0.0111
A0A0C4DH68	IGKV2-24	Immunoglobulin kappa variable 2-24	-0.5	0.0188
P01023	A2M	Alpha-2-macroglobulin	-0.5	0.0110
P03952	KLKB1	Plasma kallikrein	-0.6	0.0152
P02753	RBP4	Retinol-binding protein 4	-0.6	0.0097
P01782	IGHV3-9	Immunoglobulin heavy variable 3-9	-0.6	0.0063
P06727	APOA4	Apolipoprotein A-IV	-0.6	0.0109
A0A0J9YX35	IGHV3-64D	Immunoglobulin heavy variable 3-64D	-0.6	0.0018
P02747	C1QC	Complement C1q subcomponent subunit C	-0.7	0.0285
P02766	TTR	Transthyretin	-0.7	0.0007
A0A0C4DH67	IGKV1-8	Immunoglobulin kappa variable 1-8	-0.7	0.0016
P06396	GSN	Gelsolin	-0.7	0.0001
P02751	FN1	Fibronectin	-0.7	0.0091
P68133	ACTA1	Actin, alpha skeletal muscle	-0.8	0.0158
P04196	HRG	Histidine-rich glycoprotein	-0.8	0.0002
P09871	C1S	Complement C1s subcomponent	-0.8	0.0028

P01871	IGHM	Ig mu chain C region	-0.9	0.0018
A0A0A0MS15	IGHV3-49	Immunoglobulin heavy variable 3-49	-0.9	0.0243
P68871	HBB	Hemoglobin subunit beta	-0.9	0.0003
P01860	IGHG3	Ig gamma-3 chain C region	-0.9	0.0009
P02765	AHSG	Alpha-2-HS-glycoprotein	-0.9	0.0004
P02746	C1QB	Complement C1q subcomponent subunit B	-0.9	0.0040
P01859	IGHG2	Ig gamma-2 chain C region	-0.9	< 0.0001
P00736	C1R	Complement C1r subcomponent	-1.0	0.0077
Q06830	PRDX1	Peroxiredoxin-1	-1.0	0.0203
P05160	F13B	Coagulation factor XIII B chain	-1.0	0.0060
P48740	MASP1	Mannan-binding lectin serine protease 1	-1.1	0.0067
P02745	C1QA	Complement C1q subcomponent subunit A	-1.1	0.0005
P00488	F13A1	Coagulation factor XIII A chain	-1.1	0.0003
P00739	HPR	Haptoglobin-related protein	-1.1	0.0002
Q8WWZ8	OIT3	Oncoprotein-induced transcript 3 protein	-1.2	0.0052
P03951	F11	Coagulation factor XI	-1.3	0.0001
Q9Y6R7	FCGBP	IgGFc-binding protein	-1.4	0.0333
P69905	HBA1	Hemoglobin subunit alpha	-1.4	< 0.0001
Q15485	FCN2	Ficolin-2	-1.5	< 0.0001
P06312	IGKV4-1	Ig kappa chain V-IV region	-3.0	< 0.0001
