# Supplementary material for: Circulating microvesicles and exosomes in small cell lung cancer by quantitative proteomics
Source: Clin Proteomics. 2022 Jan 7;19:2. doi: 10.1186/s12014-021-09339-5 (PMC8903681; doi:10.1186/s12014-021-09339-5)
Supplement: Supplementary file 6 — Additional file 6: Table S5. Potential diagnostic proteins. [file 12014_2021_9339_MOESM6_ESM.pdf]

Table S5. Potential diagnostic markers for 20K and 100K samples.

20K SCLC │ Control						
**Protein**	**AUC**	**95% CI**	***p*-value**	**Sensitivity (%)**	**Specificity (%)**	**Log_2 FC**
**CRP**	0.97	0.90-1.00	0.0002	94	88	3.5
**GSN**	0.93	0.85-1.00	<0.0001	92	86	-1.0
**ANPEP**	0.91	0.80-1.00	0.0006	89	100	3.3
**TGFBI**	0.90	0.81-1.00	<0.0001	83	91	-1.3
**HBD**	0.87	0.76-0.97	<0.0001	78	83	-1.7
**HBB**	0.84	0.73-0.95	<0.0001	79	75	-1.7
**SAA1**	0.83	0.71-0.96	0.0001	82	82	2.4
**PGLYRP**	0.82	0.70-0.94	0.0001	71	79	-1.0
**TFRC**	0.80	0.67-0.94	0.0009	65	95	2.2
**LGALS3BP**	0.79	0.65-0.93	0.0006	83	78	2.2
**100K SCLC │ Control**						
**IGKV4-1**	0.98	0.95-1.00	<0.0001	96	92	-3.0
**FCN2**	0.90	0.82-0.99	<0.0001	78	92	-1.5
**F11**	0.90	0.80-1.00	0.0004	89	83	-1.3
**HBA1**	0.88	0.77-0.99	<0.0001	87	88	-1.4
**SAA2**	0.89	0.76-1.00	0.0011	85	100	3.3
**HP**	0.88	0.77-0.98	<0.0001	78	83	1.2
**CFHR4**	0.82	0.67-0.97	0.0022	81	77	1.2
**SAA1**	0.84	0.73-0.96	<0.0001	74	79	2.9
**ANPEP**	0.84	0.71-0.97	0.0003	70	84	2.4
**F13A1**	0.82	0.69-0.96	0.0005	79	76	-1.1
